# Impact of Phosphorylation on the Physiological Form of Human alpha-Synuclein in Aqueous Solution

**DOI:** 10.1021/acs.jcim.4c01172

**Published:** 2024-10-28

**Authors:** Emile de Bruyn, Anton Emil Dorn, Giulia Rossetti, Claudio Fernandez, Tiago F. Outeiro, Jörg B. Schulz, Paolo Carloni

**Affiliations:** ‡ Jülich Supercomputing Centre (JSC), Forschungszentrum Jülich GmbH, 52425 Jülich, Germany; ¶ Department of Physics, RWTH Aachen University, 52062 Aachen, Germany; § Faculty of Biology, University of Duisburg-Essen, 45141 Essen, Germany; ∥ Computational Biomedicine (IAS-5/INM-9), Forschungszentrum Jülich GmbH, 52425 Jülich, Germany; ⊥ Department of Neurology, RWTH Aachen University, 52074 Aachen, Germany; # Max Planck Laboratory for Structural Biology, Chemistry and Molecular Biophysics of Rosario (MPLbioR, UNR-MPINAT), Partner of the Max Planck Institute for Multidisciplinary Sciences (MPINAT, MPG), Centro de Estudios Interdisciplinarios, Universidad Nacional de Rosario, S2002LRK Rosario, Argentina; @ Department of NMR-based Structural Biology, Max Planck Institute for Multidisciplinary Sciences, 37077 Göttingen, Germany; △ Department of Experimental Neurodegeneration, Center for Biostructural Imaging of Neurodegeneration, University Medical Center Göttingen, 37075 Göttingen, Germany; ∇ Max Planck Institute for Multidisciplinary Sciences, 37075 Göttingen, Germany; ● Translational and Clinical Research Institute, Newcastle University, Newcastle upon Tyne NE1 7RU, United Kingdom; ◇ JARA Brain Institute Molecular Neuroscience and Neuroimaging (INM-11), Research Centre Jülich and RWTH Aachen University, 52074 Aachen, Germany

## Abstract

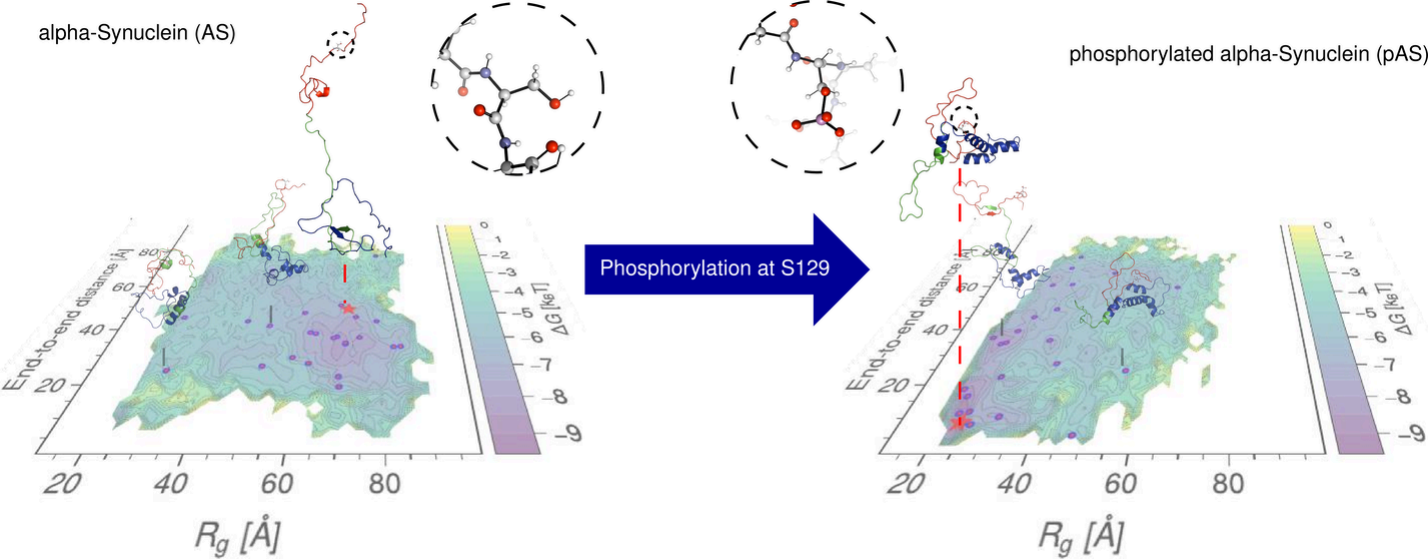

Serine 129 can be phosphorylated in pathological inclusions formed by the intrinsically disordered protein human α-synuclein (AS), a key player in Parkinson’s disease and other synucleinopathies. Here, molecular simulations provide insight into the structural ensemble of phosphorylated AS. The simulations allow us to suggest that phosphorylation significantly impacts the structural content of the physiological AS conformational ensemble in aqueous solution, as the phosphate group is mostly solvated. The hydrophobic region of AS contains β-hairpin structures, which may increase the propensity of the protein to undergo amyloid formation, as seen in the nonphysiological (nonacetylated) form of the protein in a recent molecular simulation study. Our findings are consistent with existing experimental data with the caveat of the observed limitations of the force field for the phosphorylated moiety.

## Introduction

Parkinson’s disease (PD) is the second most common neurodegenerative disease after Alzheimer’s disease,^1^ affecting several million people worldwide.^2,3^ The typical pathological hallmark is the accumulation of fibrillar protein inclusions, know as Lewy bodies (LBs) and Lewy neurites (LNs) in the brain.^4,5^ The major component of LBs and LNs is fibrillar forms of the human α-synuclein (AS) protein.^3,6^ AS is a 140 amino acid *disordered* conformational ensemble both in aqueous solution and in vivo. AS acquires some degree of structure when bound to the membrane or to cellular partners.^7,8^

**Figure 1:**
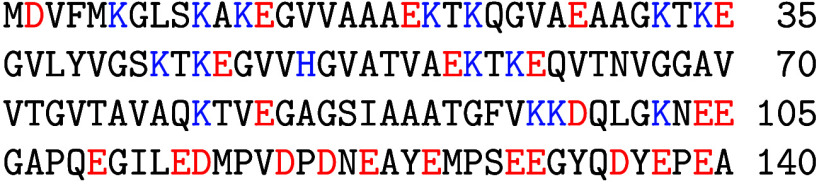
Sequence of amino acid residues in AS; positively charged residues are highlighted in blue, and negatively charged ones are highlighted in red. Three domains can be identified: the positively charged N-terminus (residues 1–60), the overall neutral hydrophobic region (residues 61–95), and the negatively charged C-terminal domain (residues 96–104). In physiological conditions, the protein is acetylated on the first residue, although this post-translational modification does not significantly affect the fibrillization propensity in vitro.^18^ In LBs, a significant fraction of AS is phosphorylated on S129.^11^ A novel phosphorylation site at T64 has also been recently described.^10^

The primary sequence of AS can be divided in three domains: the positively charged N-terminus (residues 1–60), the overall neutral hydrophobic region (residues 61–95)[We choose to use the more accurate term “hydrophobic region” instead of the historical but inaccurate term “non-amyloid component (NAC)”.^9^], and the negatively charged C-terminal domain (residues 96–140, Figure 1). We choose to use the more accurate term “hydrophobic region” instead of the historical but inaccurate term “non-amyloid component (NAC)”.^9^ Under physiological conditions, the protein is acetylated on the first residue. N-Terminal acetylation does not significantly change the fibrillization propensity in vitro.^10^ In LBs, a significant fraction of AS is phosphorylated on S129.^11^ Another phosphorylation site of α-synuclein at T64 has also been described.^10^ S129 phosphorylation may be regulated by neuronal activity, suggesting that the process may be part of the normal physiology of AS.^12,13^ This post-translational modification (PTM) might play also a pathological role.^14−17^ S129 phosphorylation may be regulated by neuronal activity, suggesting that the process may be part of the normal physiology of AS.^12,13^

The formation of the S129–O–PO_3_^2–^ group at the C-terminus of phosphorylated α synuclein (pAS) instead of one of the other two domains is intriguing, because it introduces as many as two negative charges at physiological pH (the p*K*_a__1_ and p*K*_a__2_ of phosphoserine are <2 and 5.6^19^). The phosphate group is likely partially monoprotonated under physiological conditions. The effect of protonation is discussed in the Supporting Information.

The impact of phosphorylation on the structural ensemble and aggregation propensity of physiological AS is not known. Thus, far, Circular Dichroisim (CD) studies on the non-N-term acetylated form of the protein in solution show that the conformational ensemble does not change significantly upon S129 phosphorylation.^20,21^ This contrasts with findings by CD studies for phosphorylation on protein variants.^20−22^ These point to significant changes in the structural ensemble upon phosphorylation. On the detailed molecular level, replica exchange simulations based on the CHARMM36m force field^23^ point to an increase of looped secondary structure close to a β -hairpin spread throughout the hydrophobic region upon phosphorylation.^24^ However, the structure of the physiological form differs from that of the nonacetylated one (which does not exist in human cells),^25−27^ so firm conclusions on the effect of phosphorylation on endogenous AS cannot be made from these studies.

Here we investigate the impact of phosphorylation on the physiological form of AS by molecular simulation. For this study, one may face several challenges. First, the force field must be adequate to describe IDPs such as AS. The DES-Amber ff99SB,^28^ the Amber a99SB-*disp*,^29^ and CHARMM36m^23^ force fields have been tailored for IDPs;^29−31^ the last two have been successfully used for the nonacetylated form of the proteins.^29,31,32^ All of these force fields appear therefore to be well suited to study AS. Second, accurately describing a doubly charged group such as phosphate in pAS is nontrivial. Indeed, Amber^33−35^ and CHARMM^36,37^ based simulations of phosphorylated protein have at times shown artifacts.^38−41^ Therefore, we have adapted phosphate parameters from the DES-AMBER DNA force field,^42^ recently calibrated on osmotic coefficient calculations. Finally, the conformational space of the protein structural ensemble needs to be efficiently explored. Among the many methodologies used to investigate IDPs successfully,^43−52^ our predictions based on Replica Exchange with Solute Tempering 2 (REST2)^53^ enhanced sampling predictions of wild-type^54,55^ and mutants of AS^55,56^ and turned out to reproduce a variety of biophysical properties of the protein; and hence, they appear well suitable to study this problem.

Here, we present 600 ns REST2 simulations of AS and pAS based on the DES-Amber^42^ and a99SB-*disp* force-fields.^29^ We use TIP4P-D for DES-Amber, and the accompanying modified TIP4P-D water model was used for a99SB-*disp*. To the best of the authors’ knowledge, these simulations are the only ones so far (i) reporting on the physiological form of AS in explicit solvent and (ii) describing in detail the hydration properties of the phosphate, which has never been reported in previous simulation studies.^38−41,81−85^ Many molecular simulation studies, besides those in refs^29^ and,^32^ focus on the nonacetylated protein.^31,57−80^ Calculations of the protein in an implicit solvent are not reported here.

## Methods

### Molecular Simulations

#### System

The structure of the acetylated protein (AS) which best reproduced the chemical shifts in ref (86) was selected from the conformational ensemble previously reported in ref (54). The phosphorylated protein (pAS) was built by adding a phosphate group to S129 using PyMOL.^87^

AS and pAS were inserted in a water-filled dodecahedral simulation box with periodic boundary conditions and a minimum distance of 35 Å between the protein and the box edges. Na^+^ and Cl^–^ ions were added to neutralize the system and achieve a concentration of 150 mmol L^–1^. Table 1 shows the composition of the systems.

**Table 1:** Number of Atoms in the Systems Simulated Here

	Protein	Water	Sodium	Chlorine
AS	2,020	190,533	186	176
pAS	2,023	172,359	171	159

#### Force Fields

The simulations were based on (i) the DES-Amber force field^28^ and the standard TIP4P-D water model^88^ (see Table S1 for a full list of the parameters used) and (ii) the a99SB-*disp* force field^29^ and its accompanying modified TIP4P-D water model.^29^

#### Molecular Simulation Setup

Long range electrostatics were evaluated using the Particle-Mesh Ewald (PME) method,^89^ using a cutoff distance of 12 Å in real space. The van der Waals interactions featured the same cutoff. Constant temperature conditions were achieved by coupling the systems with a Nosé–Hoover thermostat^90^ at 300 K, with a time constant of 0.5 ps. Constant pressure was achieved with a Parrinello–Rahman barostat^91^ at 1 bar, with a time constant of 2 ps (Table S1). The LINCS algorithm was used for all bonds involving hydrogen atoms.^92^ The equations of motions were integrated using the md leapfrog algorithm, with a time step of 2 fs.

#### MD and REST Simulations

The proteins underwent energy minimization (Table S2) and subsequently 100 ps of MD in the NVT ensemble (Table S3). Then, they were heated up in 25 ps-long steps of 5 K in the same ensemble up to 300 K using simulated annealing (Tables S4 and S5). The systems were further equilibrated for 1 ns in the NPT ensemble (Table S6). Finally, they underwent 600 ns REST2 simulations^53^ in the NPT ensemble, with a total of 32 replicas between 300 and 500 K exchanging every 1,000 simulation steps. The proteins were not found to be near their periodic images at distances of less than 12 Å during any of these simulations. The simulations converged after 100 ns (see the Results section).

Structurally similar conformational clusters were obtained following the method for clustering IDPs described in ref (93). For both AS and pAS, a total of 5,000 frames from the last 500 ns were clustered (Figures S7 and S8).

#### Calculated Properties

Based on the last 500 ns REST simulations, we obtained representative structures (using the method for clustering IDPs in ref (93), Figures S7 and S8), and we calculated the following properties: (i) The radius of gyration *R*_*g*_, calculated using the MDTraj Python code.^94^ (ii) The hydrodynamic radius, calculated from the radii of gyration using the linear fit of ref (67). (iii) The protein end-to-end distance between the N- and C-termini, using the MDTraj Python code.^94^ (iv) The NMR chemical shifts of backbone nitrogen, hydrogen, C_α_, C_β_, and backbone carbonyl carbon atoms, using the SPARTA+ code.^95^ (v) The CD spectra of representative cluster structures, using the SESCA code.^96−98^ (vi) The solvent accessible surface area (SASA) using the MDTraj code.^94^ (vii) The contact map of protein residues using minimum pairwise distances between residues using the MDTraj code.^94^ (viii) Radial distribution functions (RDFs) and time-resolved radial distribution functions (TRRDFs) using the SPEADI^99,100^ code developed by the authors.

(ix) Hydrogen bonds were defined according to the scheme in ref (101). (x) Salt bridges were defined using a distance between two charged atoms in the protein at a distance below 3.25 Å as in ref (102). (xi) Secondary structure elements were identified using MDTraj^94^ and DSSP.^103^ (xii) Free energy profiles (or potentials of mean force, PMFs) were calculated according to ref (104) by constructing a 2-dimensional histogram of the radius of gyration and end-to-end distance of the protein along the converged part of the simulation and subsequently performing a Boltzmann inversion of the histogram
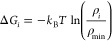
1where *ΔG*_*i*_ is the free energy at a point relative to the least dense part of the surface, and ρ_*i*_ is the density at that point.

#### Validation of the REST2 Setup

To investigate the impact of our REST2 setup parameters on our results, we performed additional 60 ns simulations with higher replicas (64) and temperatures ranging between 300 and 600 K. Comparison with 60 ns with our setup (32 replicas and temperatures ranging from 300 and 500 K) shows that these new simulations explore less efficiently the protein conformational space. Thus, increasing the number of replicas and the maximum temperature does not lead to an improvement of the results. A rationale for this result is provided in the SI on p 8 and with Figures S1 and S2.

## Results and Discussion

We performed REST2 simulations^53^ for 600 ns, using 32 replicas, for both AS and pAS in aqueous solution. Figures S3 and S4 provide details of the exchange between replicas. The root mean-square deviation (RMSD) of the simulations demonstrates that the lowest temperature replica was not trapped in local minima (Figure S5). The calculations were based on the DES-Amber^42^ and the a99SB-*disp* force fields;^29^ both were already used for IDPs. We report results at length for calculations using the former, while we provide a summary for the latter here and details in the Supporting Information.

### Convergence

We calculated two quantities as a function of simulated time to investigate the convergence of the systems (Figure S6): (i) the running averages of the percentage of secondary structures. In particular, helix structures reached a plateau after 100 ns; (ii) the running averages of the C_α_ chemical shifts which converge closely to the experimental values within 100 ns. Because of the limitations of the standard usage of RMSD with IDPs such as AS,^105^ the running RMSD of atomic positions was not taken into account beyond monitoring the simulations. Based on this analysis, we calculated all properties in the interval 100–600 ns.

### Comparison with Experiment

Experimental data was compared to properties calculated from the trajectories after the determined convergence of 100 ns.

**Figure 2:**
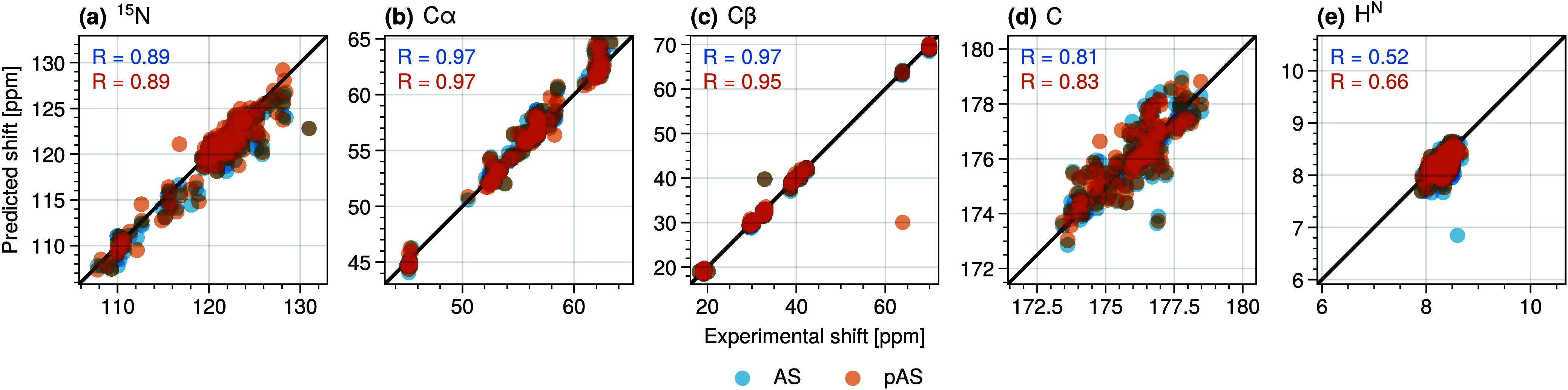
Calculated chemical shifts of (a) N, (b) C_α_, (c) C_β_, (d) C, and (e) H atoms and in AS and pAS against the experimental data from Roche et al.^86^ Correlation coefficients are given for AS (blue) and pAS (orange), respectively.

For each residue in the protein, the chemical shifts of backbone nitrogen, hydrogen, C_α_, C_β_, and backbone carbonyl carbon atoms were calculated where present in the structure. Comparison was made with the experimental values published in ref (86) (Figure 2).

The calculated chemical shifts of ^15^N, as well as the ^13^C NMR chemical shifts of C_α_ and C_β_ are in excellent agreement with the experimental values, both for AS and pAS. The calculated shifts for the backbone carbonyl carbon atoms correlate less well with those obtained through experiment yet are still broadly comparable. The calculated shifts for heavy atoms overall are in better agreement than those reported previously by some of the authors in ref (54), possibly because a much longer exploration of the conformational ensemble has been covered here (a total of 90 ns of REST2 simulations in ref (54) and 500 ns REST2 simulations here). The predicted shifts for ^1^H NMR are generally less accurate, a well-known weakness of current chemical shift prediction methods,^106^ and was previously observed in calculated values from MD simulations of AS in ref (54).

**Figure 3:**
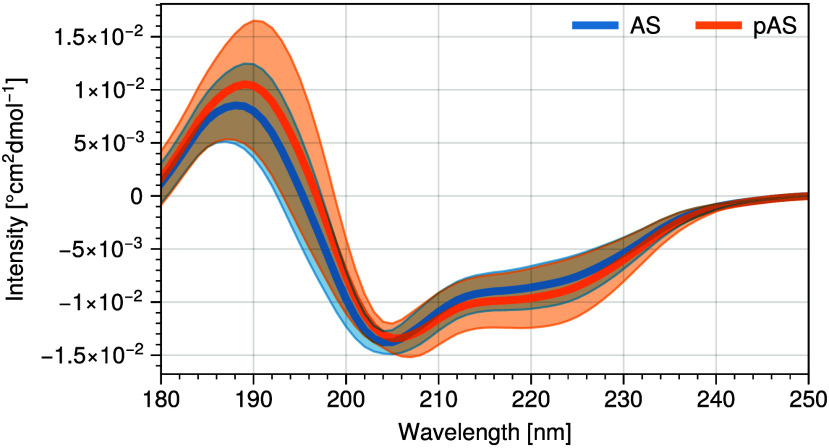
Circular Dichroism spectra of the AS and pAS cluster midpoint structures obtained during the converged part of the simulations. Shading indicates the standard error.

The calculated CD spectra of AS and pAS are in fair accord with experimentally measured spectra^27^ (Figure 3). The minima of the calculated spectra are shifted 6 nm higher than the experimentally obtained values (204 and 198 nm, respectively), similar to what was found in ref (54).

The minima of the calculated spectra range up to −15 × 10^–3°^ cm^2^ dmol^–1^ for specific conformations, similar to what was found by the authors previously. As to be expected given the improvement in force fields, these minima average to −14 × 10^–3°^cm^2^ dmol^–1^ for both AS and pAS, in much better agreement with the experimental results compared to previous results using the Amber ff99SB-*ildn* force field and TIP3P water model.^54^ The minima in the experimental spectra of Maltsev et al. are found at −19 × 10^–3°^cm^2^ dmol^–1^.

### Effect of Phosphorylation on the Protein

**Figure 4:**
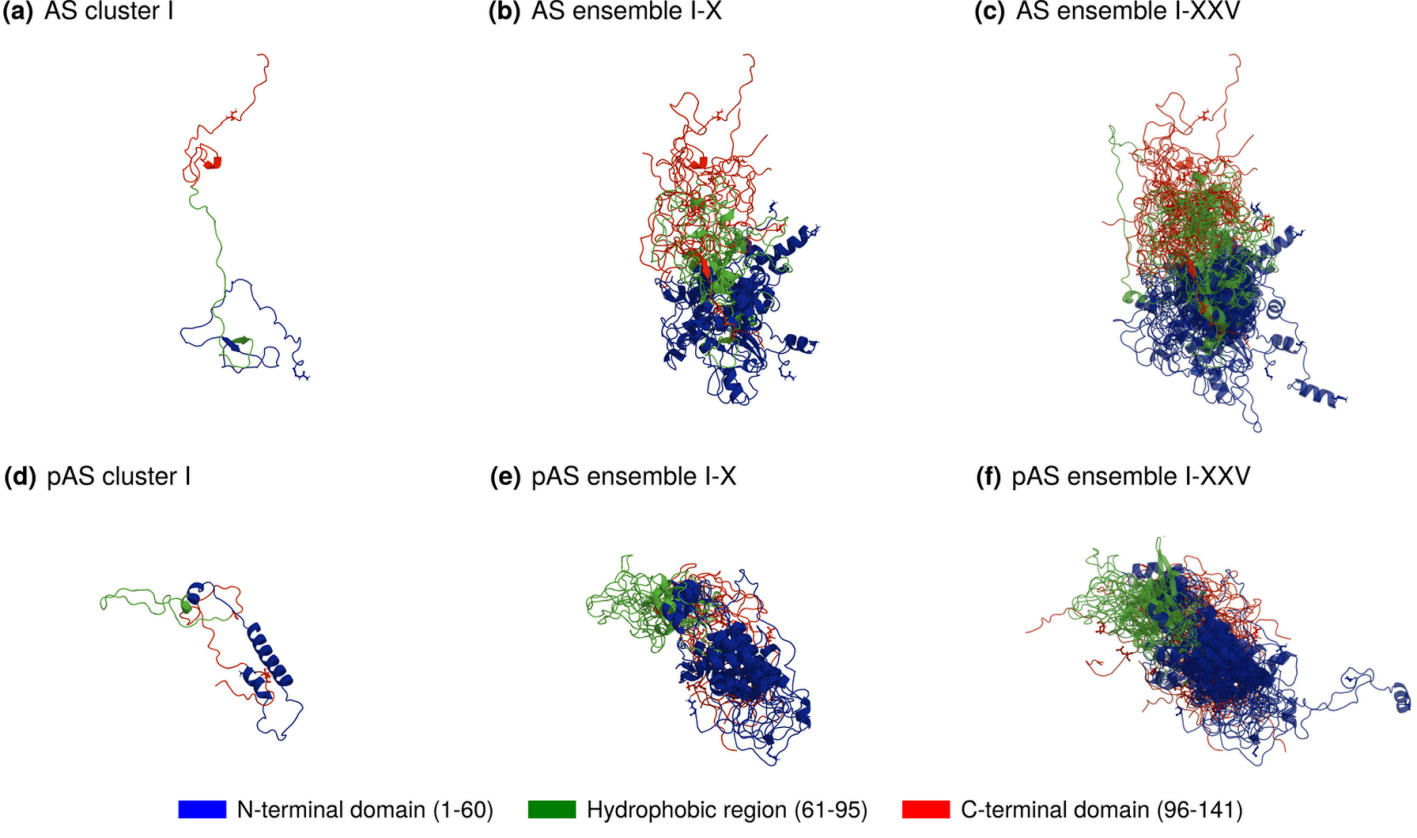
Structures of cluster midpoints representing the structural ensembles of AS (a-c) and pAS (d-f), from (a) 6.80% to (b) 48.85%, (c) 100%, (d) 5.80%, (e) 49.63%, and (f) 100%. See Table S7 for details.

The AS ensemble, on average, is less compact than the pAS ensemble (Figure 4). In AS, the C-terminal domain is further from the N-terminal domain, with the hydrophobic region situated between them. Phosphorylation increases the number of contacts between the C- and N-terminal domains, causing the hydrophobic region to shift to the side of the protein.

The ten largest conformational clusters of AS and pAS (from I to X in Figure 4) represent a total of 48.85% and 49.63% of the converged simulation trajectories, respectively (Table S7). The single conformational clusters are displayed in Figures S9 and S10.

**Figure 5:**
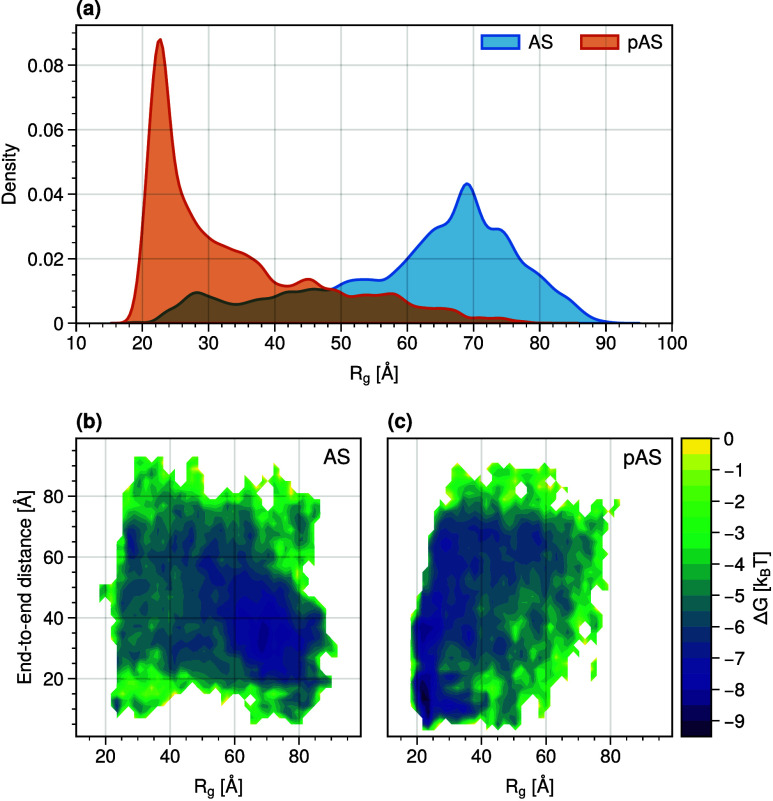
AS (blue) and pAS (orange) *R*_*g*_ distributions (a) and corresponding approximate free energy landscapes over the distance between the protein termini and radii of gyration (b-c).

The calculated mean hydrodynamic radius (*R*_*H*_) and the mean radii of gyration (*R*_*g*_) decrease significantly upon phosphorylation. The distribution of *R*_*g*_ of AS is broader than that of pAS (Figure 5(a)). The first properties (within the standard deviation) agree with experiment (28.2 and 35.3 Å for AS and pAS,^22^ respectively, Table 2).

**Table 2:** Calculated Properties of AS and pAS with Standard Deviation: (i) Hydrodynamic *R*_*H*_ and Gyration (*R*_*g*_) Radii of the Protein, (ii) Average Number of Hydrogen Bonds, and (iii) Average Number of Salt Bridges

Protein	*R*_*H*_ [Å]	*R*_*g*_ [Å]	*N*_*SB*_	*N*_*HB*_
AS	43.9 ± 21.2	61.3 ± 15.2	3.13 ± 2.23	19.52 ± 4.28
pAS	34.0 ± 19.0	33.5 ± 12.8	3.78 ± 2.64	20.67 ± 4.32
Mean change	–9.9	–27.8	0.65	1.15

An approximate estimate of the potential of mean force (PMF, see Methods), as a function of the radius of gyration and end-to-end distance of the protein, provides qualitative insights into the change in the free energy landscape of the protein upon phosphorylation. Figure 5(b-c) shows that the system passes from the shallow multibasin landscape of AS (Figure 5(b)), to the bivariate-like basin distribution for pAS (Figure 5(c)). This qualitative comparison suggests that phosphorylation induces a clear-cut separation between extended and compact ensembles of conformations for AS.

**Figure 6:**
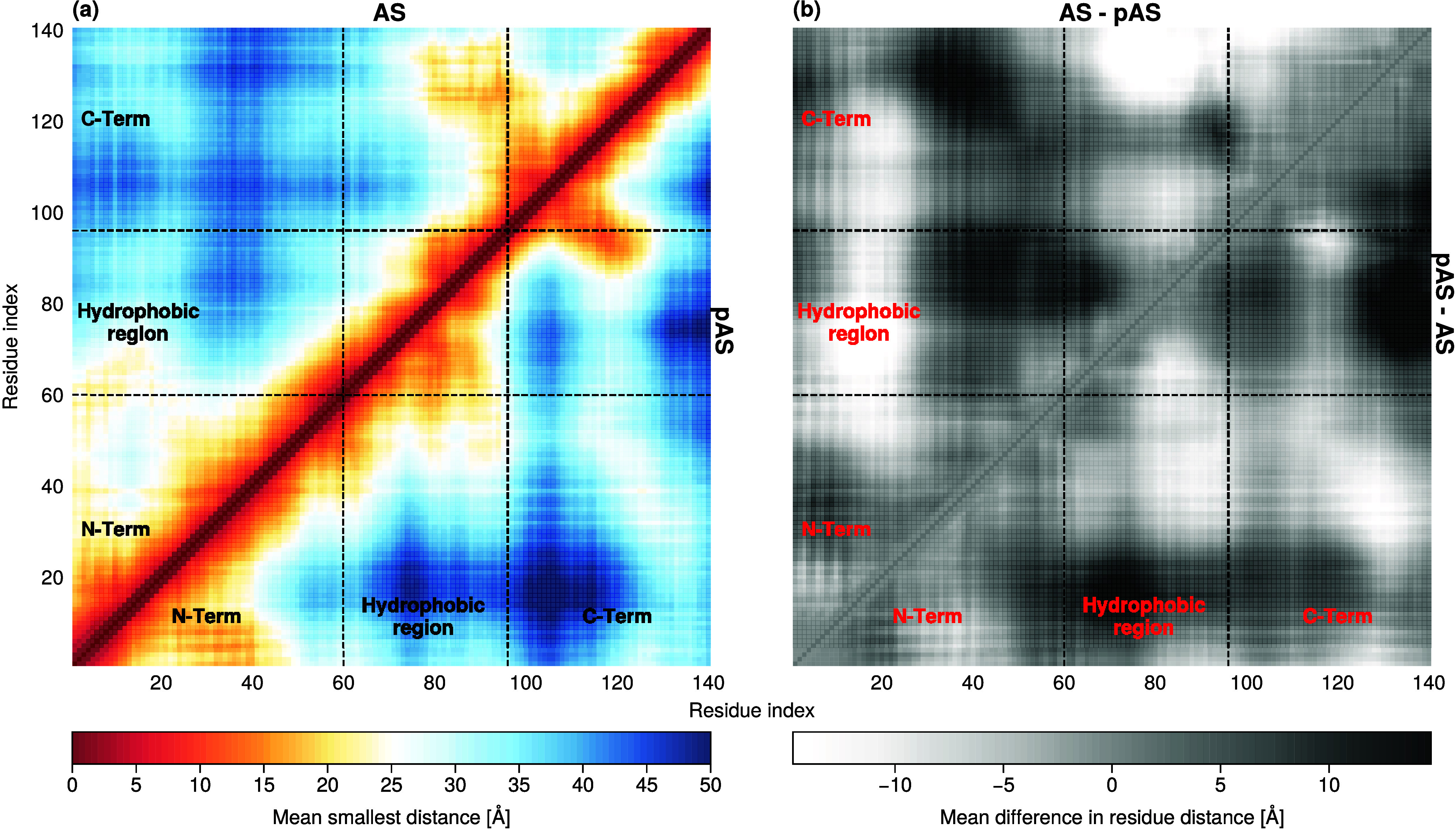
(a) Contact maps of AS (triangle above) and pAS (triangle below) and (b) their differences. Brighter values correspond to closer distances in the corresponding triangle compared to the opposite triangle.

The intramolecular interactions between the hydrophobic region and the C-terminus decrease upon phosphorylation; the C-terminus instead interacts with the N-terminal domain (Figure 6). The first dozen residues interact with the hydrophobic region in AS, while they interact with the N-terminal region in pAS (Figure 6(b)).

**Figure 7:**
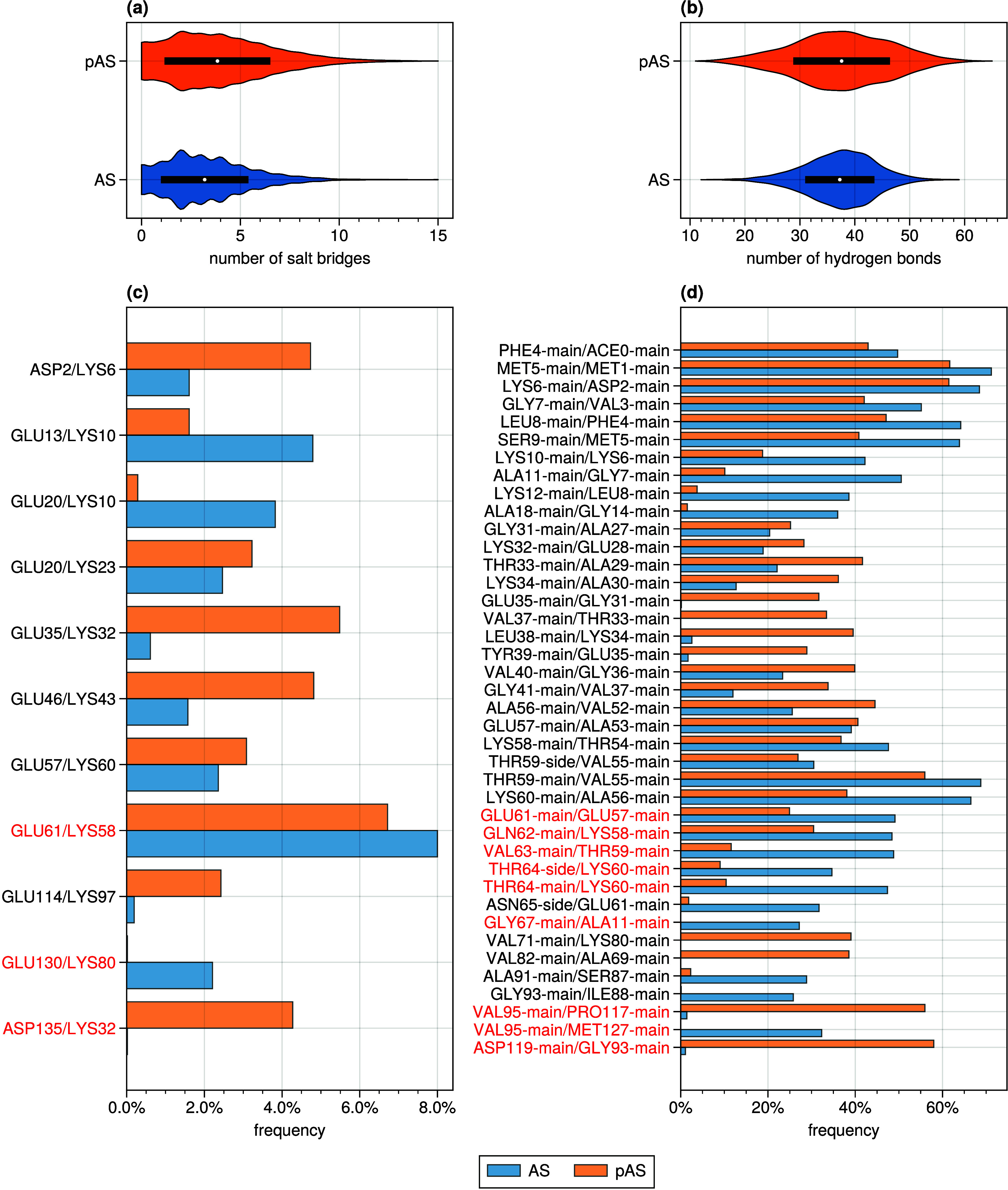
Distribution of the total number of salt bridges (a) and hydrogen bonds (b) in AS (blue) and pAS (orange). Frequency with which intradomain (black labels) and interdomain (red labels) salt bridges (c) and hydrogen bonds (d) are found in AS and pAS. Salt bridges and hydrogen bonds are displayed that occur during at least 2 and 25% of the converged trajectory, respectively, in either the AS or pAS simulation.

**Figure 8:**
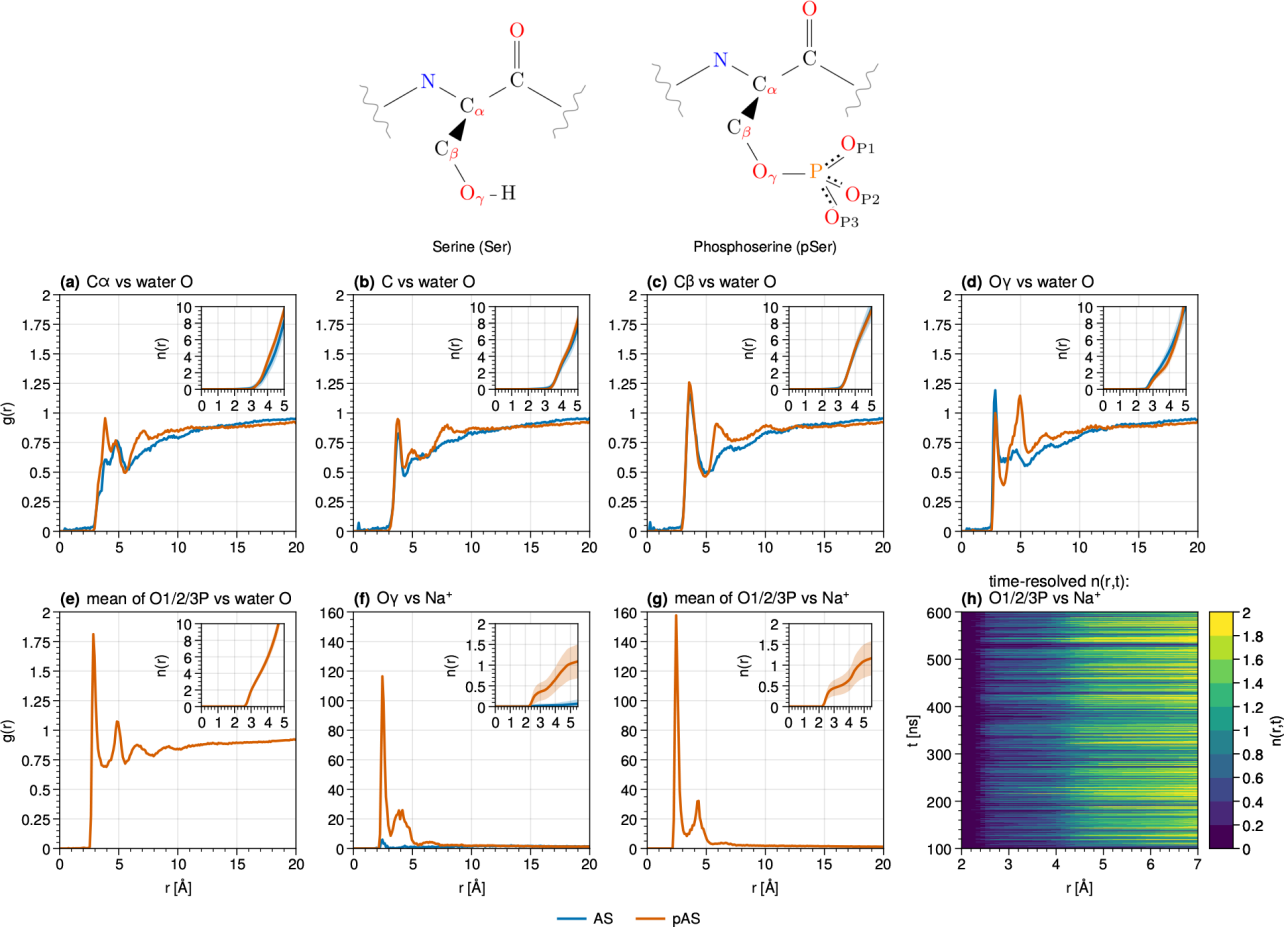
RDFs (*g*(*r*)) of (a-e) water oxygen atoms surrounding (a-b) backbone carbon atoms, (c) side-chain carbon, and (d-e) side-chain oxygen atoms. (f-g) RDFs of sodium ions surrounding side-chain oxygen atoms. (h) Integral of the TRRDF (*n*(*r*,*t*)) of sodium ions over 1 ns time windows. Insets show the integral of *g*(*r*) up to 5 Å.

**Figure 9:**
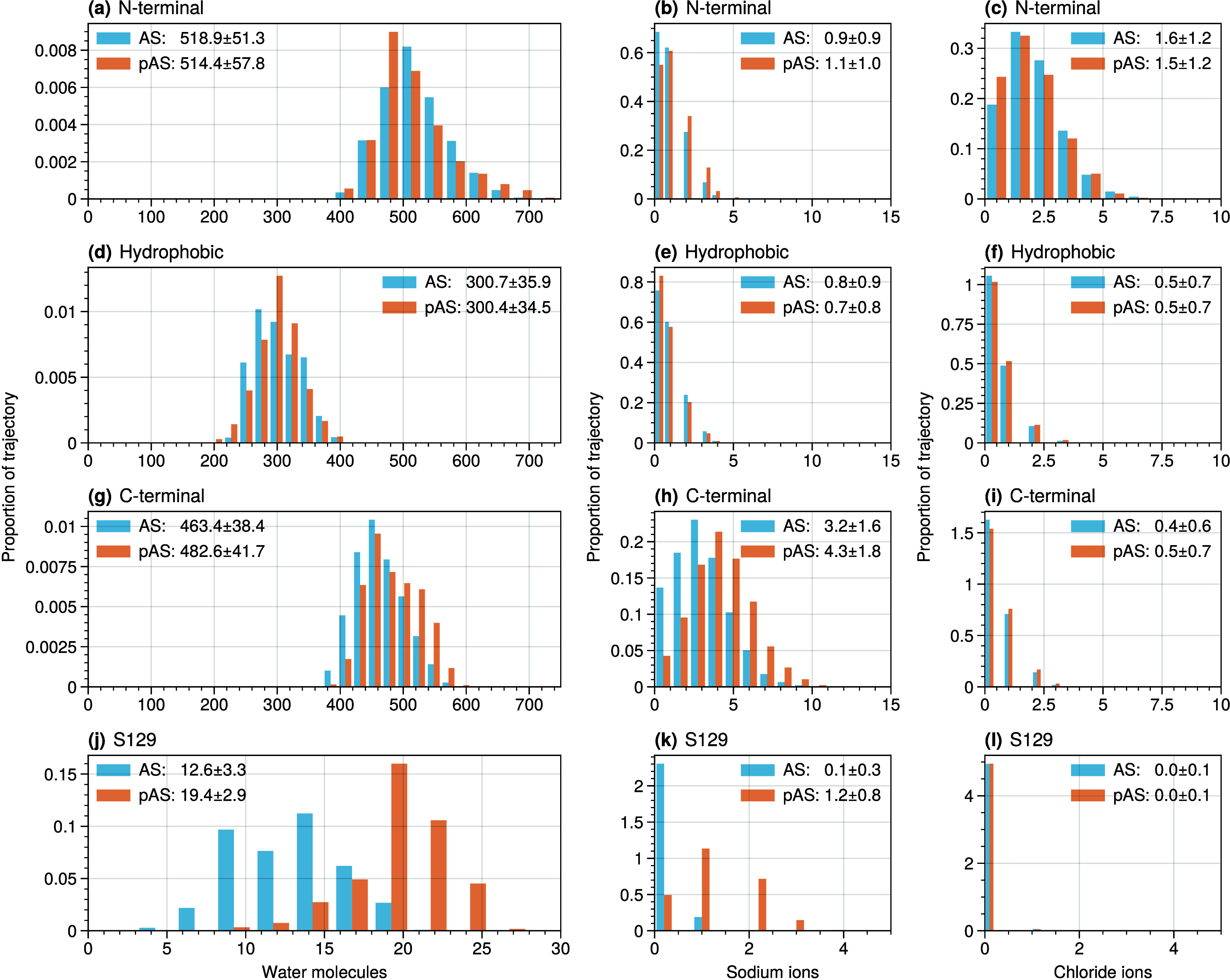
Number of water molecules (left column), sodium ions (middle column), and chloride ions (right column) in the first hydration shell surrounding the (a-c) N-terminal, (d-f) hydrophobic, and (g-i) C-terminal domains of the protein, as well as around (j-l) the S129 residue. Inset numbers indicate the mean and standard deviation of the distributions.

The number of hydrogen bonds and salt bridges within their standard deviations does not change significantly upon phosphorylation (Figure 7, Table 2). While the first are almost exclusively formed within every single domain (Figure 7(a-b)), persistent salt bridges are formed for both proteins between the N-term and hydrophobic regions (K23–E20 and K58-E61, respectively; Figure 7(c)). Few salt bridges are formed between the C-terminal domain and one of the two other domains, such as E130–K80 in AS. The absence of the E130–K80 salt bridge in pAS might be caused by the presence of sodium counterions close to the pS129 residue (Figures 8 and 9).

The Average Solvent Accessible Surface Area (SASA) decreases upon phosphorylation (Table 3). However, the decline is rather small (within the standard deviation) at the N-term and in the hydrophobic region.

**Table 3:** Calculated SASA in the Three Domains of AS and pAS

Protein	SASA_N-term_ [Å^2^]	SASA_HydrophR_ [Å^2^]	SASA_C-term_ [Å^2^]
AS	6112.4 ± 603.1	3478.1 ± 585.6	6952.6 ± 762.4
pAS	5732.3 ± 726.3	3104.0 ± 472.0	6035.0 ± 955.3
Mean change	–380.1	– 374.1	–917.6

### Phosphate Interactions

The phosphate group is fully solvent-exposed and associated with sodium counterions without interactions with pAS residues (Figures 8 and 9). Thus, its electrostatic field is strongly reduced, and its long-range electrostatic interactions with the C-terminus and N-terminus are expected to be strongly screened (Figure 7).

The S129 side-chain in AS is less hydrated than that in pAS: while the Oγ atom is surrounded on average by two water molecules in the first hydration shell in both AS and pAS, the second and third hydration shells contain many more water molecules in pAS (Figure 8). The serine oxidril group in AS instead forms a variety of *intramolecular* H-bonds (with K80, K96, K97, K102, E126, E130 and E131; Figure S19). S129 backbone units are observed to interact with both the solvent and nearby protein hydrogens in both AS and pAS. Thus, S129 in AS forms many more intramolecular contacts than the corresponding phosphorylated residue in pAS.

The hydration of AS N-terminal and hydrophobic domains is comparable to that of pAS (Figure 9(a-b)). The hydration of the C-terminal domain instead increases upon phosphorylation, possibly because of the presence of the highly charged group (from 12.3(3.3) water molecules surrounding S129 to 19.5(2.7) around pSer129).

### Additional Simulations

The simulations of AS and pAS with the Amber a99SB-*disp* force field^29^ show very similar results as those presented here, except for the phosphate hydration properties, which turn out to be less accurate than those of the DES-Amber force field (See Supporting Information, Sections 4, 5).

The simulations of the protein with monoprotonated phosphate (based on the a99SB-*disp* force field) turn out to be rather similar to those of pAS (see Supporting Information, Section 6). Thus, we conclude that if such species exist in equilibrium with pAS, they contribute to the protein structural ensemble similarly to pAS.

### Role of Phosphorylation for AS Fibril Formation

Our study in line with experimental studies shows that phosphorylation and dephosphorylation of AS are likely normal physiological processes fine-tuning binding to lipids,^107,108^ and they are not a clear marker of pathology.^12,13^ These findings, however, do strengthen the prevailing view that phosphorylation of the monomer is also implicated in fibril formation, due to the change in the structural ensemble and relative positioning of the domains. In addition, the content of the β-hairpin-like structure in the hydrophobic region (calculated as in ref (24)) turns out to increase upon phosphorylation (Figure S23). This content is however smaller than that observed for the nonphysiological form, see details in the Supporting Information. As discussed in ref (24), these types of structures may be associated with amyloid-forming conformations, and hence, this finding does suggest that phosphorylation increases fibril formation starting from conformations similar to those found in the fibrils.^109,110^ This might be consistent with the fact that almost all the proteins in the fibrils are phosphorylated *in vivo*.^11^

## Conclusions

Because of the presence *in vivo* of phosphorylated AS, a detailed understanding of the impact of phosphorylation on this protein is important for informing therapeutic strategies aimed at targeting AS in synucleinopathies. Here, we investigated the effects of phosphorylation on the structural ensemble of AS in solution by 600 ns REST2 simulations based on apt force fields such as DES-Amber and Amber a99SB-*disp*. Our REST2 simulations of AS, much longer than the previously reported ones,^54^ are consistent with a plethora of experimental data.The physiological form of pAS turns out to be more compact than the unmodified protein. The phosphate moiety is solvent exposed without forming specific intramolecular interactions. The phosphorylation of the protein turns out to induce β-hairpin-like, amyloid-forming conformations. The increased propensity toward fibril formation might be consistent with the fact that about 90% AS in the LBs is phosphorylated.^11^

## Data Availability

GROMACS 2022.6 patched with PLUMED 2.9.0 was used to perform all MD simulations (https://www.gromacs.org/ and https://www.plumed.org/). All analysis employing third-party software are described and referenced in the Methods section. RDFs were obtained using the authors’ open-source Python package SPEADI (https://github.com/FZJ-JSC/speadi and https://pypi.org/project/SPEADI/). Charts and plots were made using the open-source Python package ProPlot (https://github.com/proplot-dev/proplot). Molecular structures were visualized using Open-Source PyMOL (https://github.com/schrodinger/pymol-open-source). Primary data available to reproduce the study (parametrized GROMACS topologies, input files, and trajectories) are deposited in Zenodo: https://zenodo.org/records/12605636.

## Supporting Information Available

Detailed experimental setup (including all GROMACS parameters), analysis and comparison with results obtained with other force fields (PDF)
Structures of the 25 cluster midpoints are included for both AS and pAS (ZIP)
