# Supplementary material for: Impact of Phosphorylation on the Physiological Form of Human alpha-Synuclein in Aqueous Solution
Source: J Chem Inf Model. 2024 Oct 28;64(21):8215–26. doi: 10.1021/acs.jcim.4c01172 (PMC11558680; doi:10.1021/acs.jcim.4c01172)
Supplement: Supplementary file 1 — ci4c01172_si_002.pdf [file ci4c01172_si_002.pdf]

Supplementary Information for Impact of Phosphorylation on the Physiological Form of Human alpha-Synuclein in Aqueous Solution

Emile de Bruyn,[†,‡,¶] Anton Emil Dorn,[†,‡,§] Giulia Rossetti,[*,‡,‖,⊥] Claudio Fernandez,[#,@] Tiago F. Outeiro,[△,∇,††] Jörg B. Schulz,[¶,⊥,‡‡] and Paolo Carloni[¶,‖]

† *Equally contributed to this work*
‡ *Jülich Supercomputing Centre (JSC), Forschungszentrum Jülich GmbH, 52425 Jülich, Germany*
¶ *Department of Physics, RWTH Aachen University, 52062 Aachen, Germany*
§ *Faculty of Biology, University of Duisburg-Essen, 45141 Essen, Germany*
‖ *Computational Biomedicine (IAS-5/INM-9), Forschungszentrum Jülich GmbH, 52425 Jülich, Germany*
⊥ *Department of Neurology, RWTH Aachen University, 52074 Aachen, Germany*
Max Planck Laboratory for Structural Biology, Chemistry and Molecular Biophysics of Rosario (MPLbioR, UNR-MPINAT). Partner of the Max Planck Institute for Multidisciplinary Sciences (MPINAT, MPG). Centro de Estudios Interdisciplinarios, Universidad Nacional de Rosario, S2002LRK Rosario, Argentina
@ *Department of NMR-based Structural Biology, Max Planck Institute for Multidisciplinary Sciences, 37077 Göttingen, Germany*
△ *Department of Experimental Neurodegeneration, Center for Biostructural Imaging of Neurodegeneration, University Medical Center Göttingen, 37075 Göttingen, Germany*
∇ *Max Planck Institute for Multidisciplinary Sciences, 37075 Göttingen, Germany*
†† *Translational and Clinical Research Institute, Newcastle University, Newcastle upon Tyne NE1 7RU, United Kingdom*
‡‡ *JARA Brain Institute Molecular Neuroscience and Neuroimaging (INM-11), Research Centre Jülich and RWTH Aachen University, 52074 Aachen, Germany*

E-mail: g.rossetti@fz-juelich.de

1 Simulation parameters

Tables S1 to S6 show the parameters used to initiate the different runs in GROMACS. Tables S2 to S6 describe both the preparation for the simulations using REST2 and the unbiased MD simulations. The parameters contained in table S1 were used for each replica during the REST2 simulation.

Table S1: Parameters used in GROMACS for the production run using REST2 and unbiased MD. Unbiased production runs used the same parameters.

Parameter	Value
integrator	md
nsteps	300,000,000
dt [ps]	0.002
nstxout	250
nstvout	250
nstenergy	250
nstlog	5000
nstxout-compressed	0
nstcalcenergy	250
compressed-x-grps	Protein, Ion
energygrps	Protein, Water, Ion
constraint_algorithm	lincs
constraints	h-bonds
lincs_iter	1
lincs_order	4
cutoff-scheme	Verlet
nstlist	10
coulombtype	PME
pme_order	4
rlist [nm]	1.2
rcoulomb [nm]	1.2
rvdw [nm]	1.2
fourierspacing [nm]	0.12
tcoupl	Nose-Hoover
tc-grps	Protein, Water, Ion
tau_t [ps]	0.5
ref_t [K]	300
pcoupl	Parrinello-Rahman
pcoupltype	isotropic
tau_p [ps]	2.0
ref_p [bar]	1.0
compressibility [bar^{-1}]	4.5e-5
DispCorr	EnerPres
pbc	xyz
gen_vel	no
nstcomm	100
comm-grps	Protein, Water, Ion

Table S2: Parameters used in GROMACS for the energy minimization.

Parameter	Value
integrator	steep
nsteps	100000
emtol [kJ mol^{-1} nm^{-1}]	10
emstep [nm]	0.01
nstlog	1000
nstenergy	100
energygrps	System
cutoff-scheme	Verlet
nstlist	1
rlist	1
pbc	xyz
pme_order	4
vdwtype	Cut-off
vdw-modifier	Potential-shift-Verlet
rcoulomb [nm]	1.2
rvdw [nm]	1.2
epsilon_r [nm]	1
fourierspacing [nm]	0.12

Table S3: Parameters used in GROMACS for the NVT equilibration.

Parameter	Value
integrator	md
nsteps	50000
dt [ps]	0.002
nstxout	500
nstvout	500
nstenergy	500
nstlog	500
continuation	no
constraint_algorithm	lincs
constraints	h-bonds
lincs_iter	1
lincs_order	4
cutoff-scheme	Verlet
nstlist	10
rcoulomb [nm]	1.2
rvdw [nm]	1.2
DispCorr	EnerPres

Continued on next page

Continued from previous page

Parameter	Value
coulombtype	PME
pme_order	4
fourierspacing [nm]	0.12
tcoupl	Nose-Hoover
tc-grps	Protein, Water, Ion
tau_t [ps]	0.5
ref_t [K]	300
pcoupl	no
pbc	xyz
gen_vel	yes
gen_temp [K]	300
gen_seed	-1
Define	-DPOSRES

Table S4: Parameters used in GROMACS for the simulated annealing.

Parameter	Value
integrator	md
nsteps	550000
dt [ps]	0.002
nstxout	2000
nstvout	2000
nstenergy	1000
nstlog	1000
nstxout-compressed	5000
compressed-x-grps	System
energygrps	System
continuation	no
constraint_algorithm	lincs
constraints	none
lincs_iter	1
lincs_order	4
lincs-warnangle	30
morse	no
cutoff-scheme	Verlet
nstlist	1
coulombtype	PME
pme_order	4
vdwtype	Cut-off
vdw-modifier	Potential-shift-Verlet

Continued on next page

Continued from previous page

Parameter	Value
rcoulomb [nm]	1.2
rvdw [nm]	1.2
epsilon_r [nm]	1
fourierspacing [nm]	0.12
tcoupl	Nose-Hoover
tc-grps	System
tau_t [ps]	0.1
ref_t [K]	300
pcoupl	Parrinello-Rahman
pcoupltype	isotropic
tau_p [ps]	0.5
ref_p [bar]	1.0
compressibility [bar^{-1}]	4.5e-5
nstcomm	1
comm-mode	linear
comm-grps	Protein, Water, Ion
pbc	xyz
DispCorr	EnerPres
gen_vel	yes
gen-temp	2
gen-seed	173529
annealing	single
annealing_npoints	40
Define	-DPOSRES

Table S5: Temperature and time steps in the simulated annealing run.

annealing_time	annealing_temp
0	2
50	5
75	10
100	15
125	20
150	25
175	30
200	35
225	40
250	45
275	50
300	55

Continued on next page

Continued from previous page

annealing_time	annealing_temp
325	60
350	65
375	70
400	75
425	80
450	85
475	90
500	100
525	110
550	120
575	130
600	140
625	150
650	160
675	170
700	180
725	190
750	200
775	210
800	220
825	230
850	240
875	250
900	260
925	270
950	280
975	290
1000	300

Table S6: Parameters used in GROMACS for the NPT equilibration.

Parameter	Value
integrator	md
nsteps	500000
dt [ps]	0.002
nstxout	500
nstvout	500
nstenergy	500
nstlog	500
continuation	yes

Continued on next page

Continued from previous page

Parameter	Value
constraint_algorithm	lincs
constraints	h-bonds
lincs_iter	1
lincs_order	4
cutoff-scheme	Verlet
nstlist	10
rcoulomb [nm]	1.2
rvdw [nm]	1.2
DispCorr	EnerPres
coulombtype	PME
pme_order	4
fourierspacing [nm]	0.12
tcoupl	Nose-Hoover
tc-grps	Protein, Water, Ion
tau_t [ps]	0.5
ref_t [K]	300
pcoupl	Parrinello-Rahman
pcoupltype	semiisotropic
tau_p [ps]	2.0
ref_p [bar]	1.0
compressibility [bar^{-1}]	4.5e-5
refcoord_scaling	com
pbc	xyz
gen_vel	no
Define	-DPOSRES

1.1 Choice of REST2 Simulation Ensemble Parameters

The REST2 algorithm does not increase the temperature of the whole system. Instead it scales the forces acting between protein atoms by a *lambda* factor ($\lambda = \frac{T_0}{T_m}$, where T_0 is the lowest temperature and T_m is the temperature at *lambda* state m) and those between the protein atoms and solvent by $\sqrt{\lambda}$. Relatively large exploration on the conformational landscape is associated with large differences between the total protein-protein interaction energy and the total protein-solvent interaction energy.[1]

Additional 60 ns simulations of AS and pAS were conducted with two sets of REST2 parameters: using 32 replicas between 300 and 500 K and using 64 replicas between 300 and 600 K. Figure S1 shows a larger difference between protein-protein and protein-solvent interaction energies in the simulations using 32 replicas than that with 64 replicas.

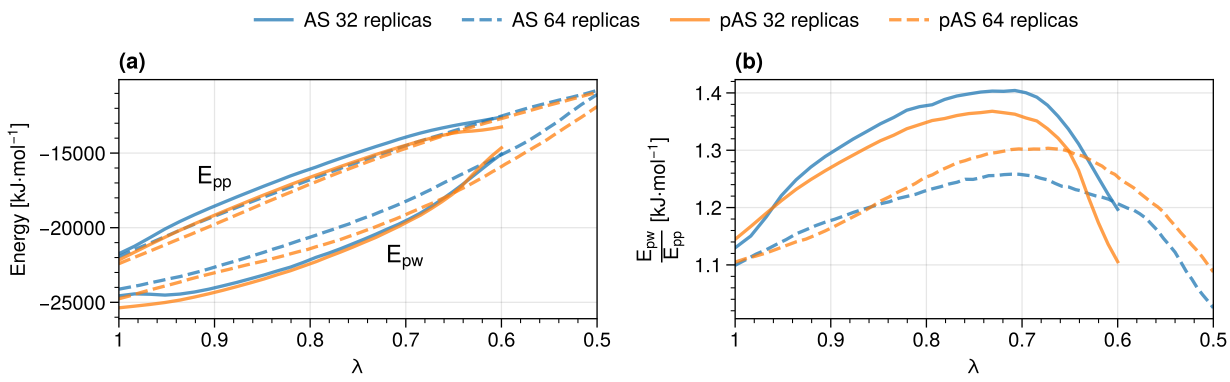

Figure S1: (a) mean protein-protein (E_{pp}) and protein-solvent (E_{pw}) interaction energies over 60 ns of simulation, and (b) the ratio between the former and latter.

The extent of exploration on the conformational landscape for the two sets of parameters illustrates the effect of a larger energy term difference (Figure S2). Despite a higher mean acceptance ratio when exchanging replicas between λ states, 31 and 32 % vs 11 and 13 % for AS and pAS respectively, the extent of exploration is smaller for the larger ensemble than for the smaller ensemble, for both AS and pAS.

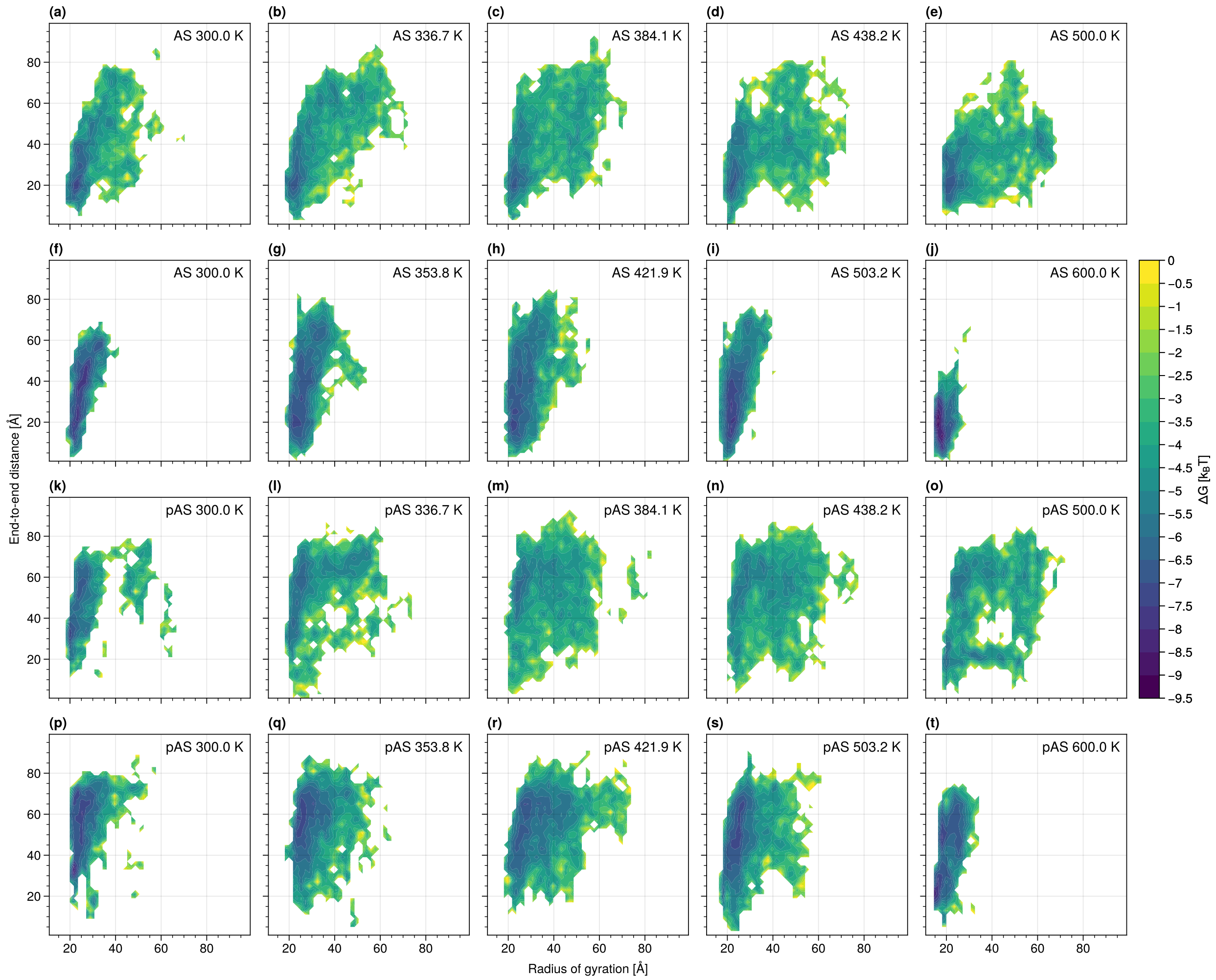

Figure S2: Comparison of the protein conformational landscape by REST2 ensemble. Conformational landscapes of (a-e) the AS simulation using 32 replicas between 300 and 500 K, (f-j) the AS simulation using 64 replicas between 300 and 600 K, (k-o) the pAS simulation using 32 replicas between 300 and 500 K, and (p-t) the pAS simulation using 64 replicas between 300 and 600 K.

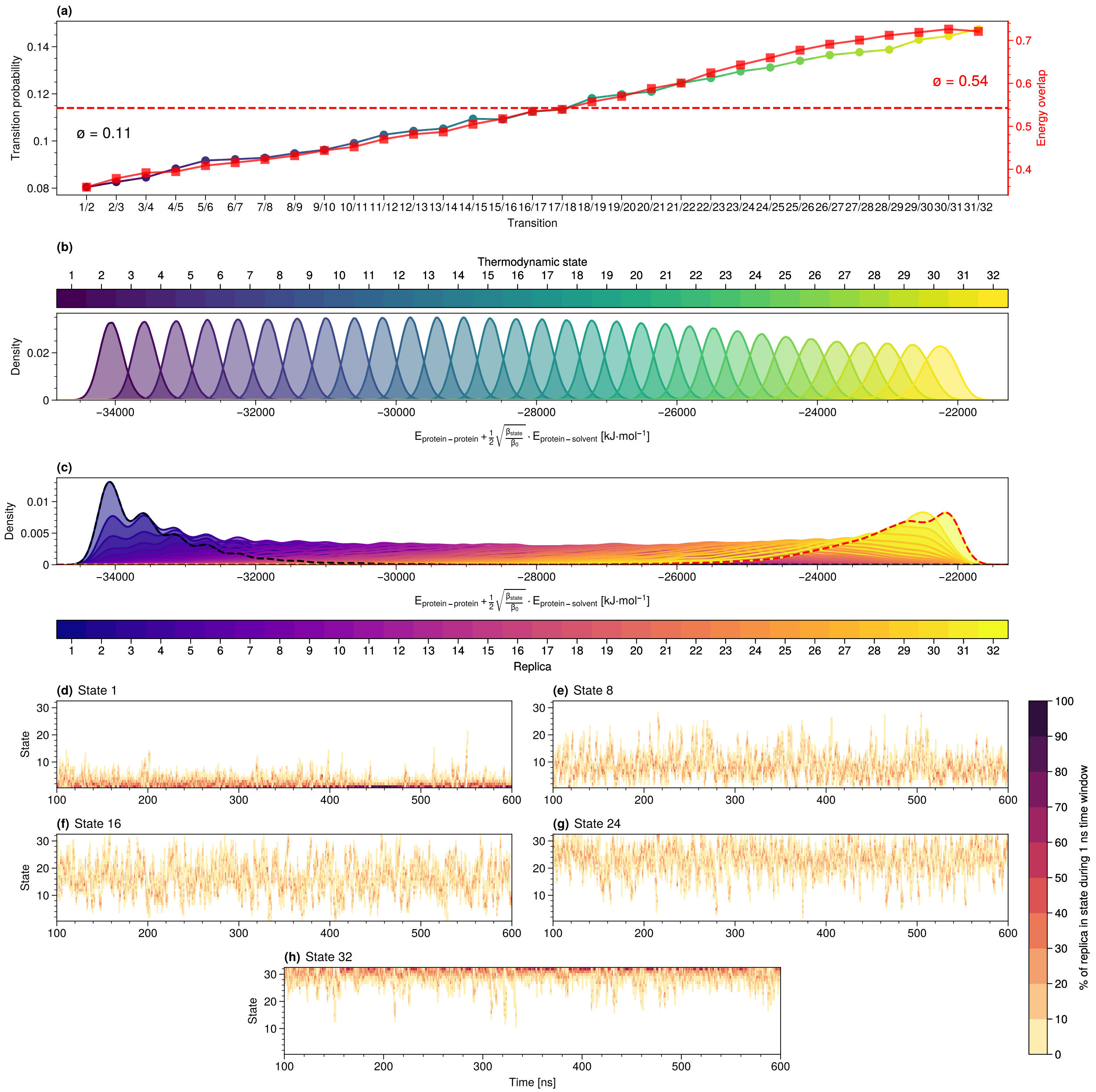

Figure S3: Replica exchange statistics for the converged simulation of AS. (a) The transition probability and energy overlaps between λ states. (b) The distribution of energies over λ states used for the acceptance criterion when exchanging replicas, and (c) the same energy distributed over the replicas. (d-h) Occupancy of selected λ states in replicas over the simulation.

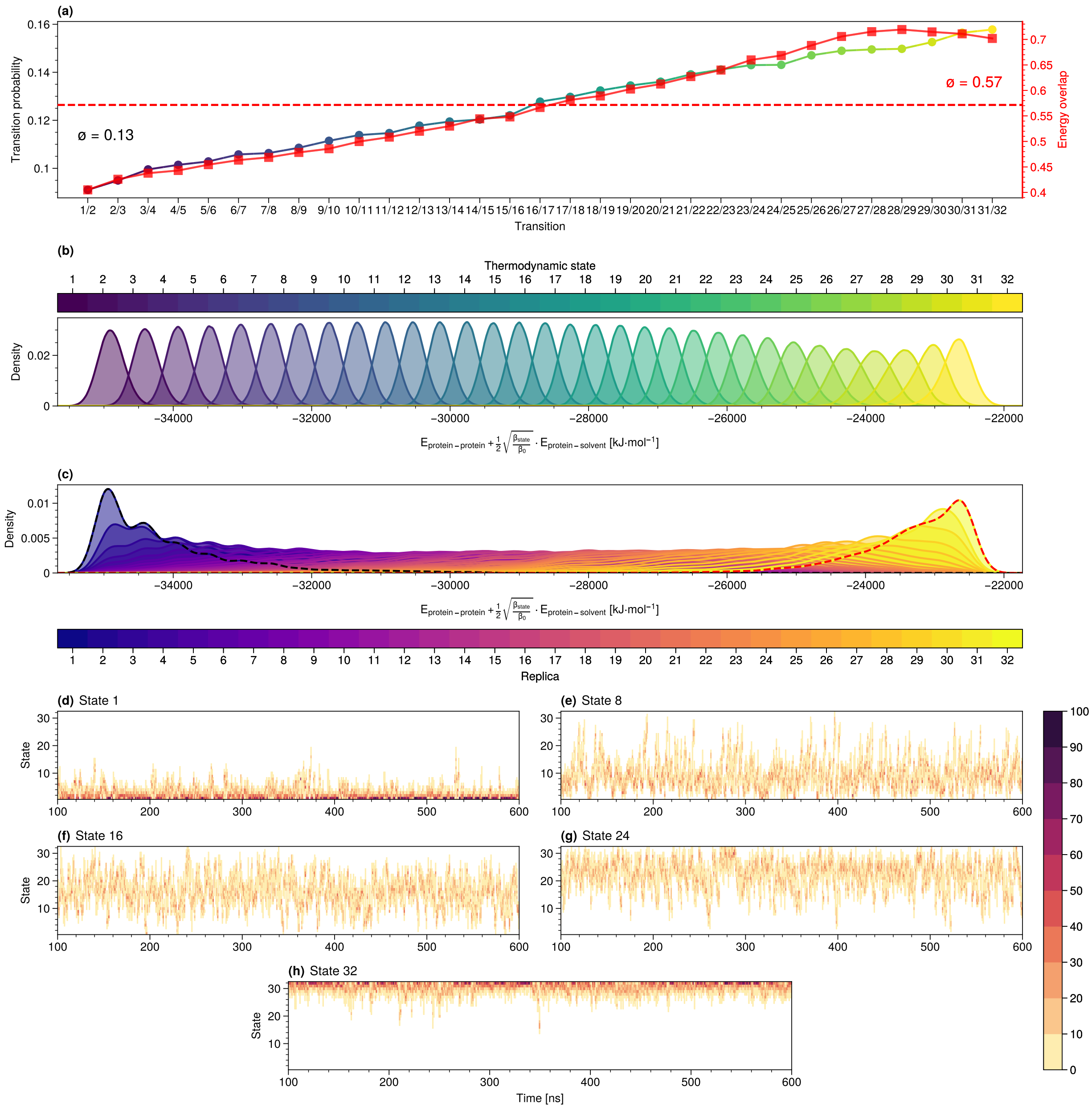

Figure S4: Replica exchange statistics for the converged simulation of pAS. (a) The transition probability and energy overlaps between λ states. (b) The distribution of energies over λ states used for the acceptance criterion when exchanging replicas, and (c) the same energy distributed over the replicas. (d-h) Occupancy of selected λ states in replicas over the simulation.

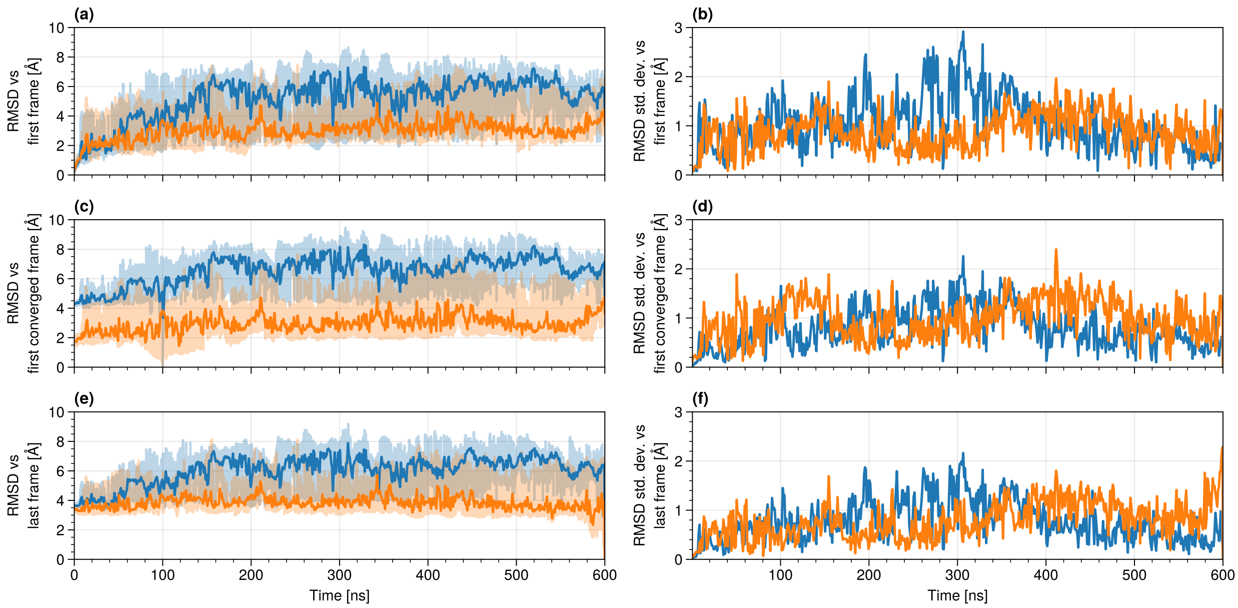

Figure S5: DES-Amber force field-based simulations: (a,c,e) RMSD of the simulation vs (a) the initial configuration, (b) the first frame of the converged part of the trajectory and (c) the last frame in the trajectory; (b,d,f) standard deviation of the RMSDs over 100 frames vs the frames in (a,c,e).

2 Convergence tests

This section investigates the convergence of the simulations. The running time averaged values of chemical shifts and secondary structure percentages for AS and pAS are reported in Figure S6(a-b) and (c-d), respectively.

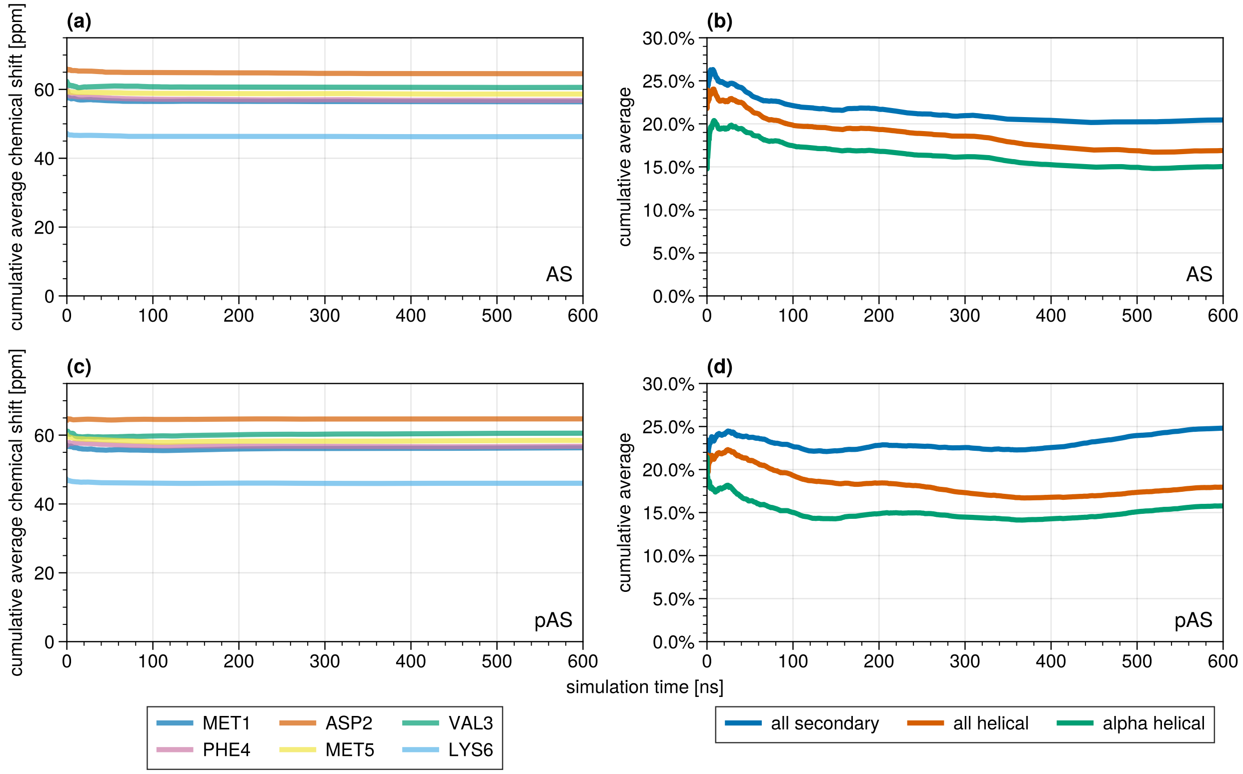

Figure S6: DES-Amber force field-based simulations: (a,c) running time averaged chemical shifts of the C_α atoms of the first six residues; (b,d) running time averaged occurrence of secondary structures (red) and helices (blue) as percent of all residues.

3 Obtaining Structurally Similar Clusters

High-dimensional pair-wise root mean-square displacement (RMSD) values between individual trajectory frames were projected onto a lower-dimensional plane using a t-distributed Stochastic Neighbor Embedding (t-SNE) approach, following the method described by Appadurai et al.[2] This projection was then K-means clustered to obtain cluster members and midpoints. t-SNE perplexity values and number of clusters K were grid-searched to optimal silhouette scores (i.e. optimal separation of clusters).

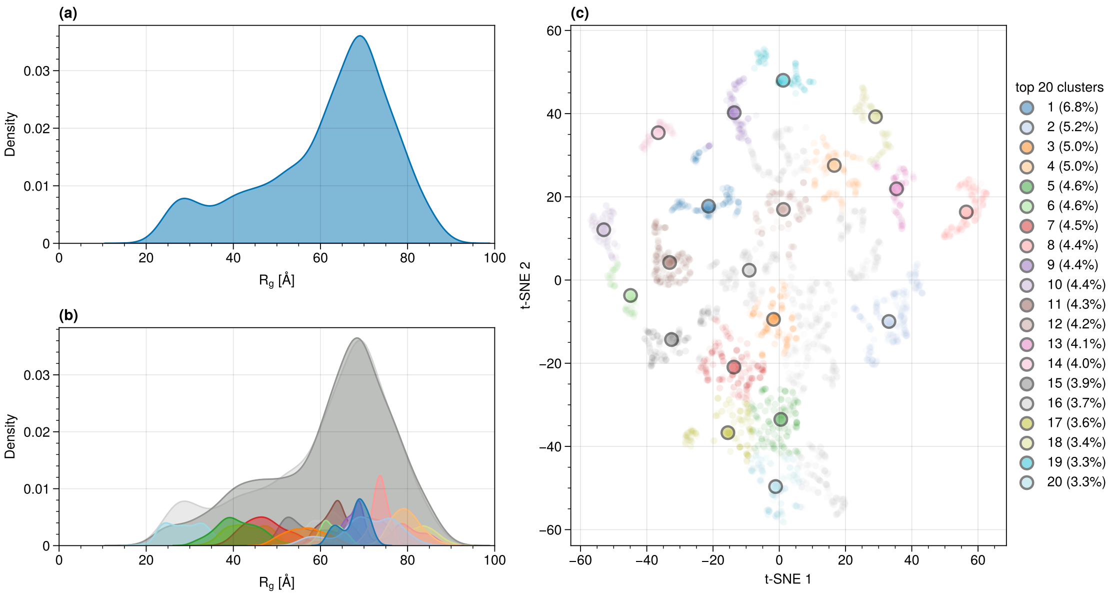

Figure S7: t-SNE clustering structures from the AS trajectory.

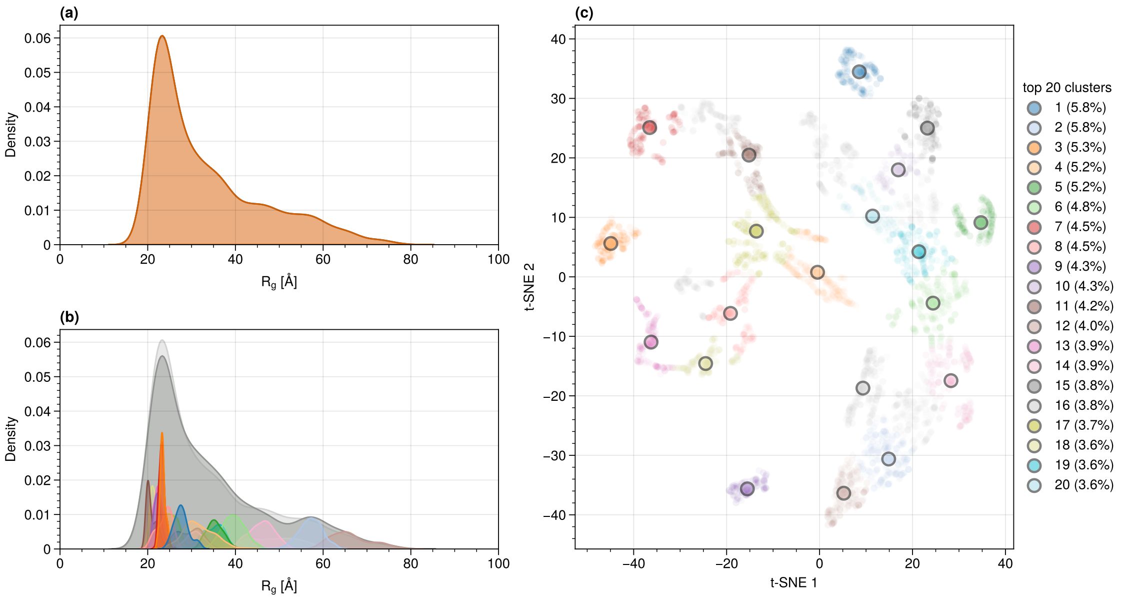

Figure S8: t-SNE clustering structures from the pAS trajectory.

Table S7: Percentage of occurrence of the ten most representative structures of unphosphorylated AS and pAS in the DES-Amber simulation.

Cluster	AS [%]	pAS [%]
I	6.80	5.80
II	5.24	5.76
III	5.00	5.30
IV	5.00	5.18
V	4.64	5.16
VI	4.56	4.84
VII	4.52	4.48
VIII	4.38	4.48
IX	4.36	4.34
X	4.36	4.30
	48.85	49.63

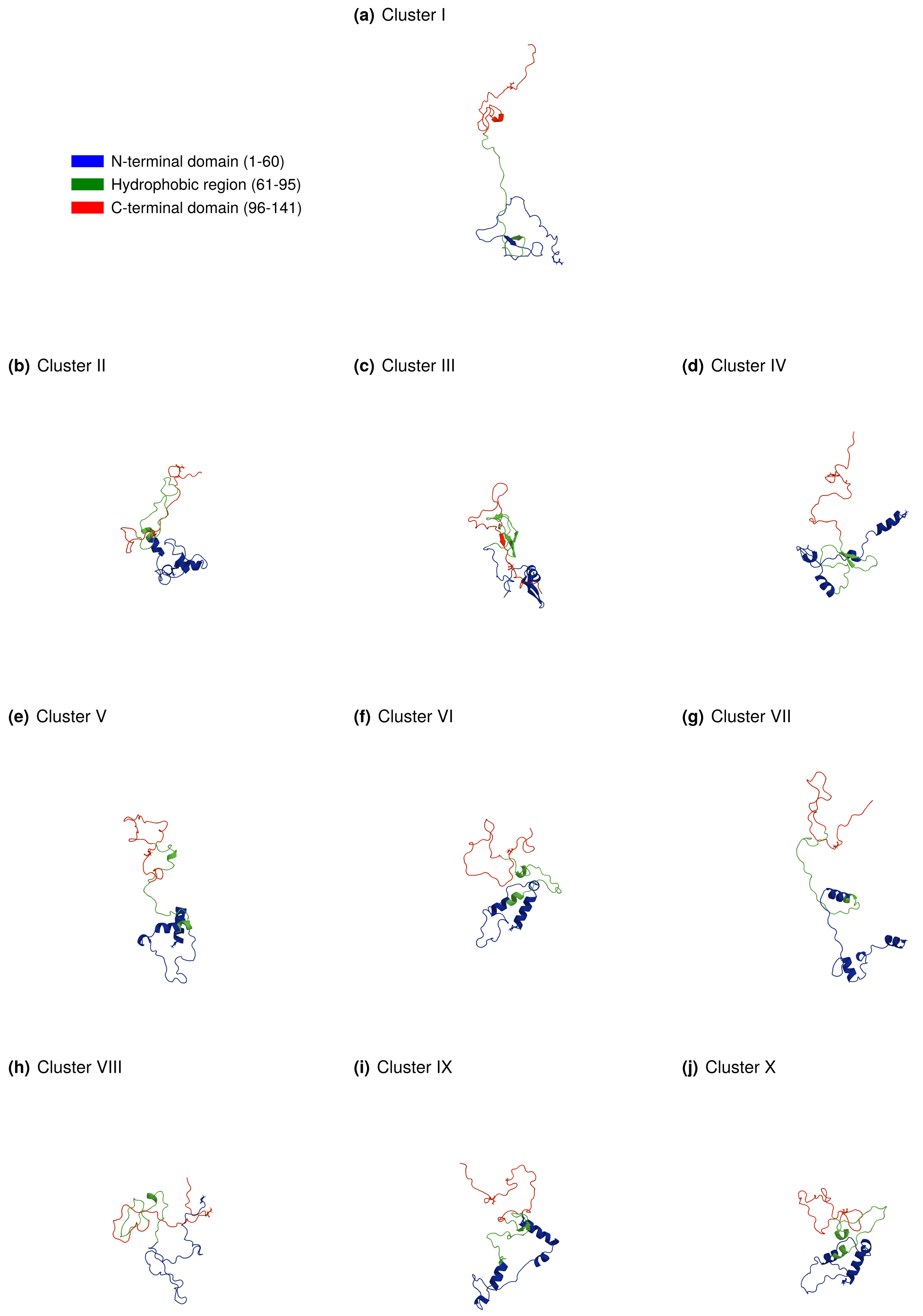

Figure S9: Representative structures of AS as determined by clustering. The N-terminal domain is colored blue, the hydrophobic region green and the C-terminal domain red. The percentage of occurrence of the structures decreases from I to X (Table S7).

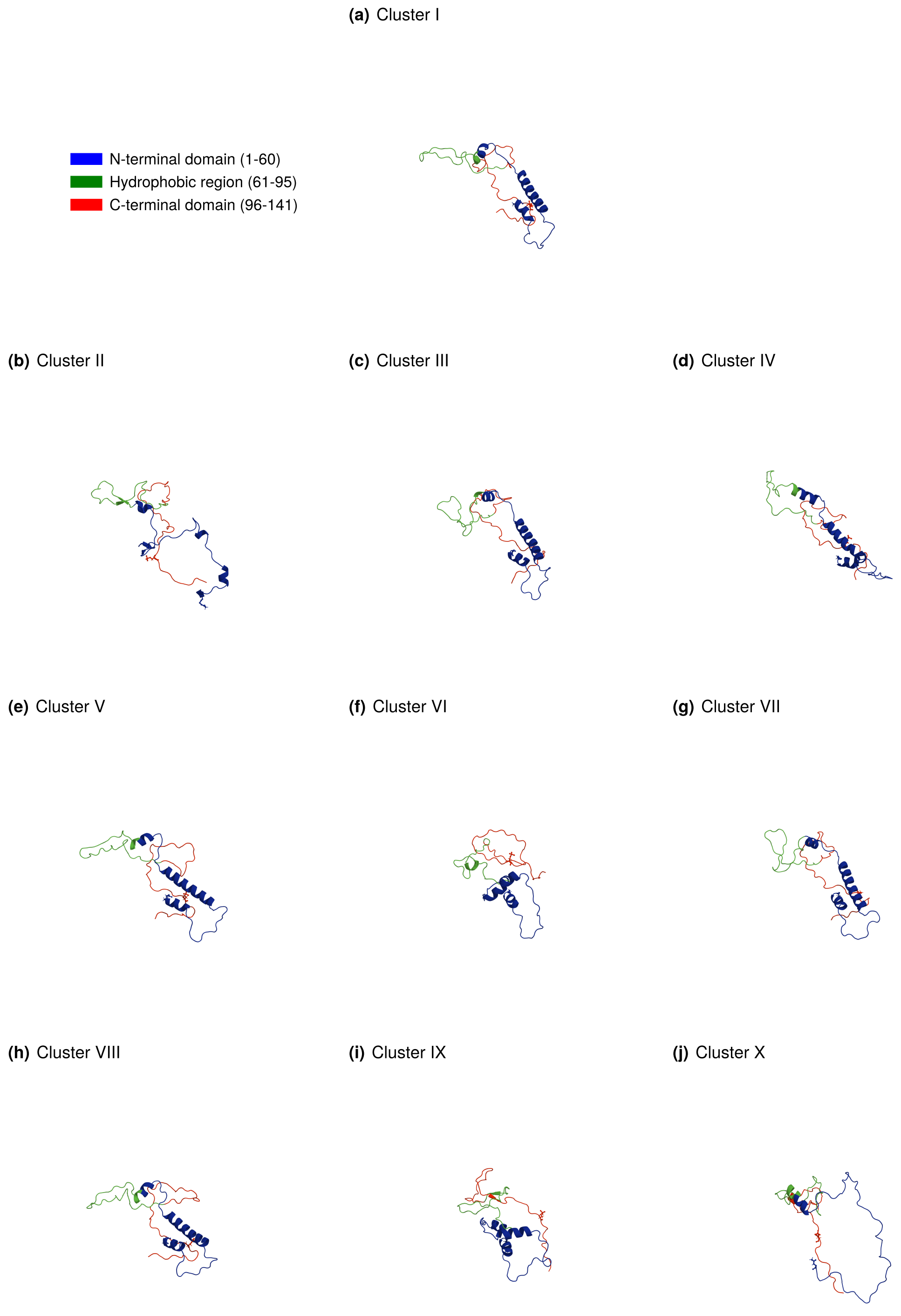

Figure S10: Representative structures of pAS as determined by clustering. The acetyl group at the N-terminus and the residue at position S129 are shown as ball and sticks. The percentage of occurrence of the structures decreases from I to X (Table S7).

4 Amber a99SB-*disp* force field results

Here we present additional data for three secondary simulations using the Amber a99SB-*disp* force field. These simulations are all 25 ns in length.

Table S8: Number of atoms in the simulation box for AS, pASH[1] and pAS.

	Protein	Water	Sodium	Chlorine
AS	2,020	68,370	75	65
pASH	2,024	79,431	86	75
pAS	2,023	68,361	77	65

Convergence tests This section investigates the convergence of the a99SB-*disp* simulations. The running time averaged values of chemical shifts and secondary structure percentages for AS, pASH and pAS are reported in Figure S11(a-b) and (c-d), respectively.

[1]In this system, the phosphate is monoprotonated.

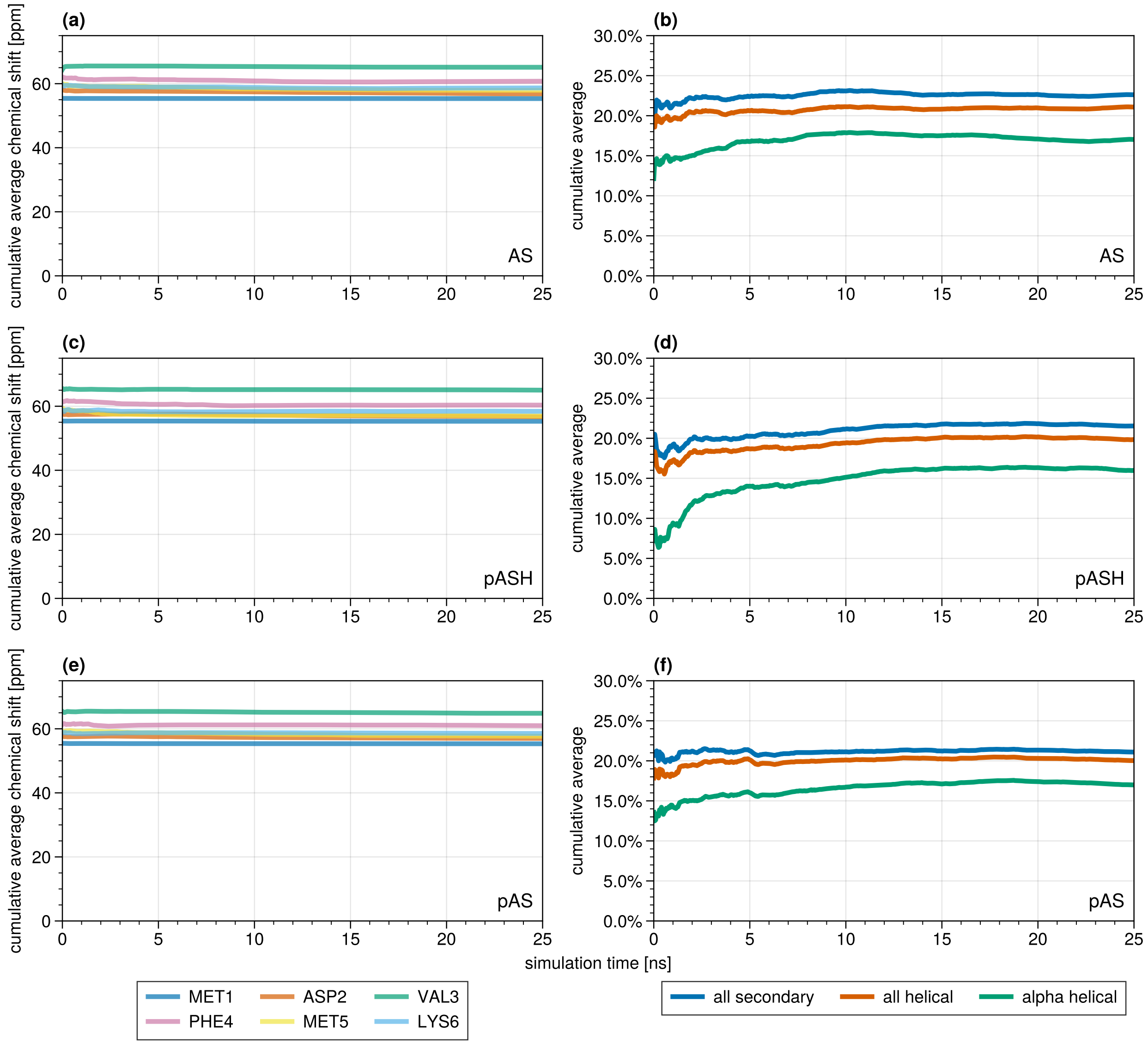

Figure S11: a99SB-*disp* force field-based simulations: (a,c,e) running time averaged chemical shifts of the C_α atoms of the first six residues; (b,d,f) running time averaged occurrence of secondary structures (red) and helices (blue) as percent of all residues.

Obtaining structurally similar clusters.

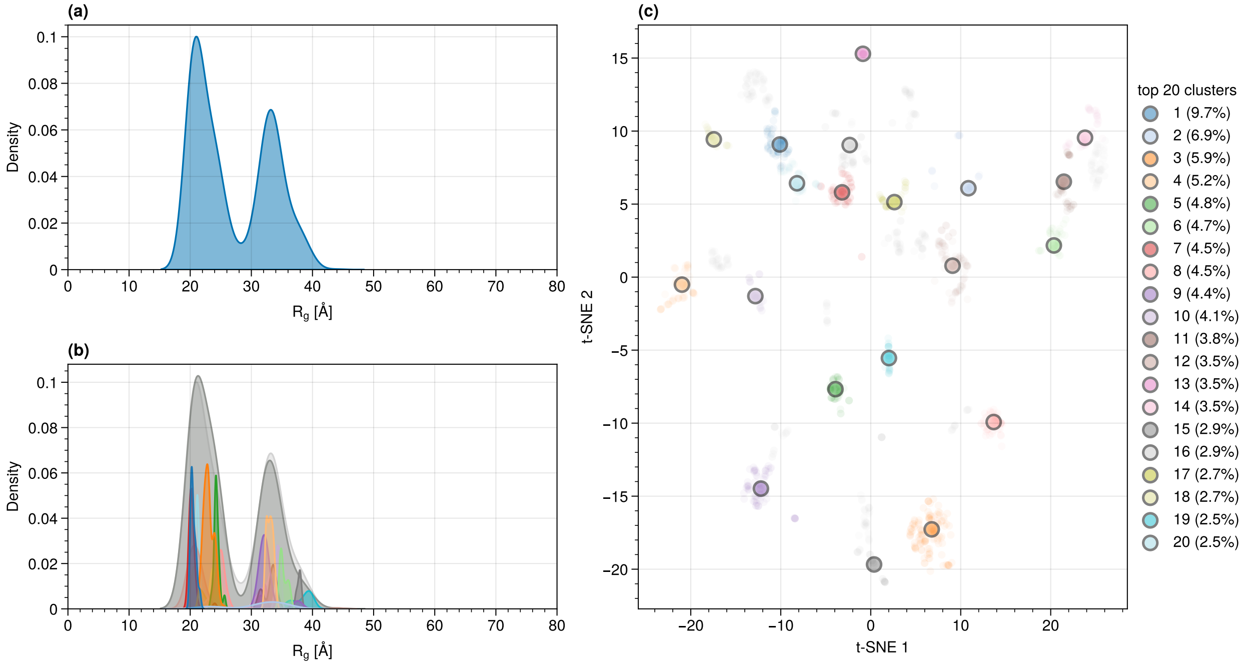

Figure S12: t-SNE clustering structures from the AS trajectory.

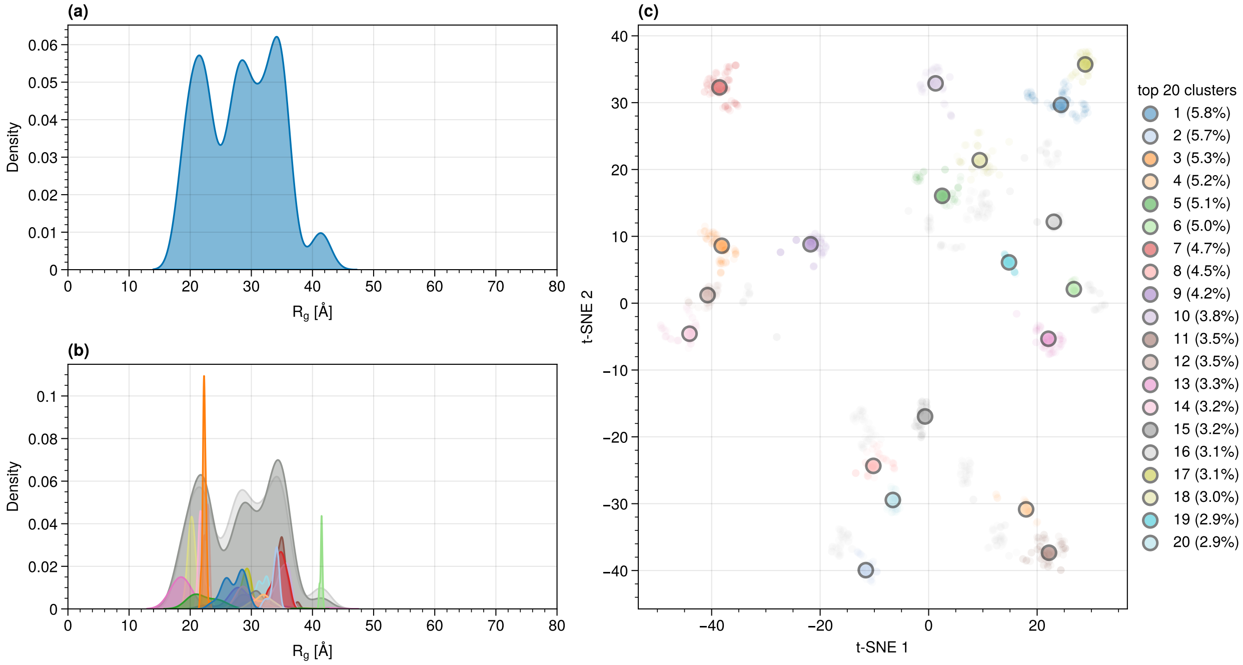

Figure S13: t-SNE clustering structures from the pASH trajectory.

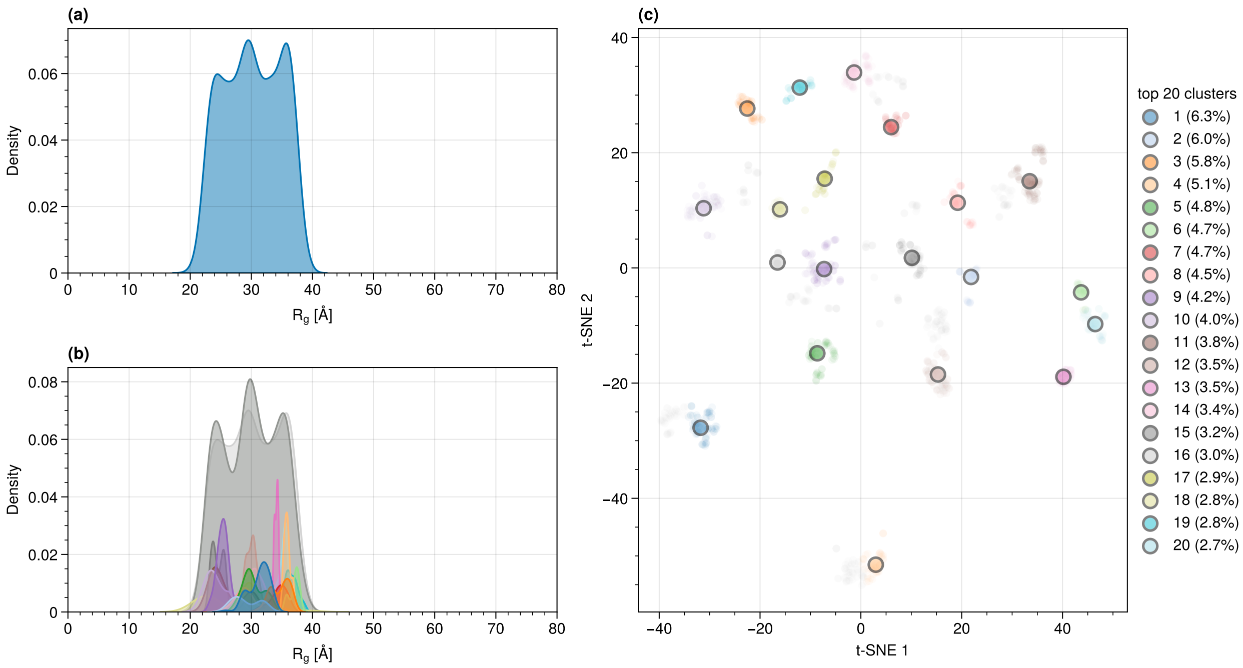

Figure S14: t-SNE clustering structures from the pAS trajectory.

Size and radius of AS upon phosphorylation.

Table S9: Calculated properties of AS, pASH and pAS. (i) Hydrodynamic radii R_H and radii of gyration (R_g) of the entire proteins and of the hydrophobic regions (HR). The experimental value of R_H is 28.7 Å.[3] (ii) Average number of hydrogen bonds. (iii) Average number of salt bridges. Standard deviations are indicated in parenthesis.

Protein	R_H [Å]	R_g [Å]	R_g(HR) [Å]	R_H(HR) [Å]	N_{SB}	N_{HB}
AS	30.8 (± 3.5)	27.6 (± 5.7)	16.7 (± 5.7)	22.8 (± 5.2)	2.86 (± 1.96)	21.51 (± 4.19)
pASH	30.9 (± 3.0)	27.8 (± 5.5)	17.4 (± 7.7)	23.3 (± 6.7)	1.77 (± 1.53)	21.02 (± 4.61)
pAS	31.8 (± 2.7)	29.3 (± 4.5)	18.7 (± 7.6)	24.4 (± 6.4)	1.85 (± 1.62)	22.13 (± 4.18)
AS (exp)	28.7					

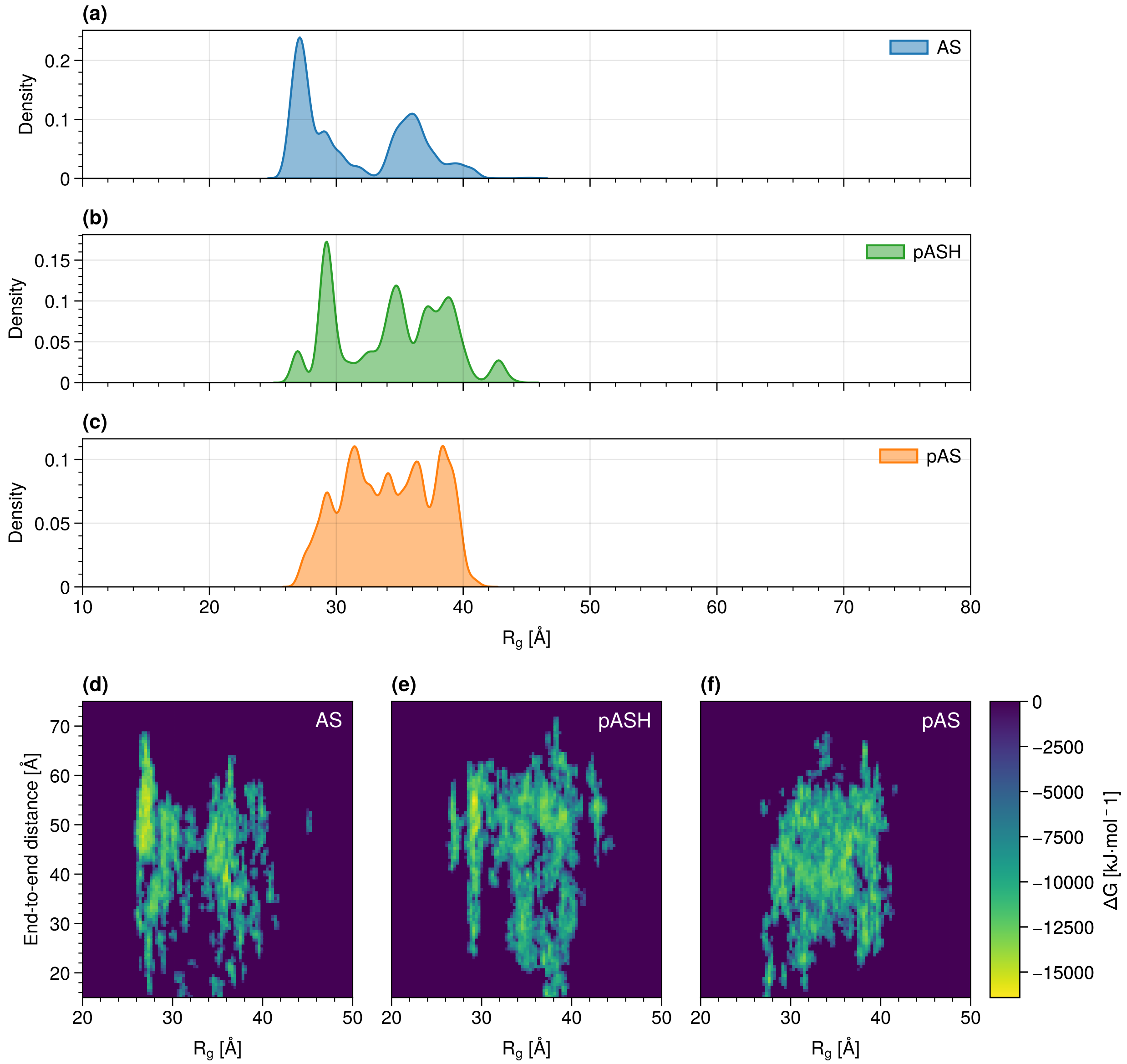

Figure S15: R_g distribution for AS (blue), pASH (green), and pAS (orange) (a-c) and the corresponding (highly approximate) free energy landscapes plotted as a function of the distance between the protein termini (d-f).

End-to-end distances. The distance from the N- to C-terminus shows no clear trend (Figure S16).

Solvent accessibility. The average solvent accessibility of the a99SB-*disp* based simulations (Table S11) closely resemble those found for the simulations based on DES-Amber (Table S11). The averages SASA values are all well within the standard deviation of each other.

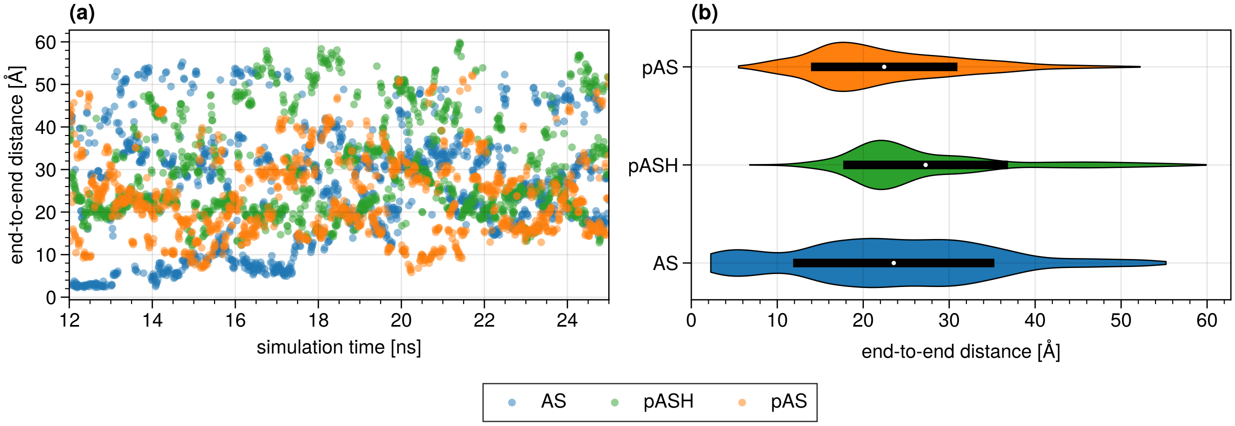

Figure S16: Distance from the N- to C-terminus for all three proteins against simulation time in the a99SB-*disp* simulation.

Table S10: N-terminus – C-terminus distances of AS, pAS, pASH averaged over the 12 ns to 25 ns simulation period in the a99SB-*disp* simulation.

Protein	Distance d [Å]	σ_d [Å]
AS	27.8	12.9
pASH	30.8	11.9
pAS	25.9	08.6

Table S11: Average solvent accessible surface areas (SASA) in AS and pAS in the hydrophobic region (HR) and N- and C-terminus using the a99SB-*disp* force field.

Protein	$SASA_N$ [Å^2]	$SASA_{HR}$ [Å^2]	$SASA_C$ [Å^2]
AS	87 (± 38)	83 (± 40)	107 (± 34)
pASH	90 (± 42)	84 (± 36)	112 (± 36)
pAS	86 (± 46)	85 (± 39)	107 (± 39)

Salt bridges and hydrogen bonds.

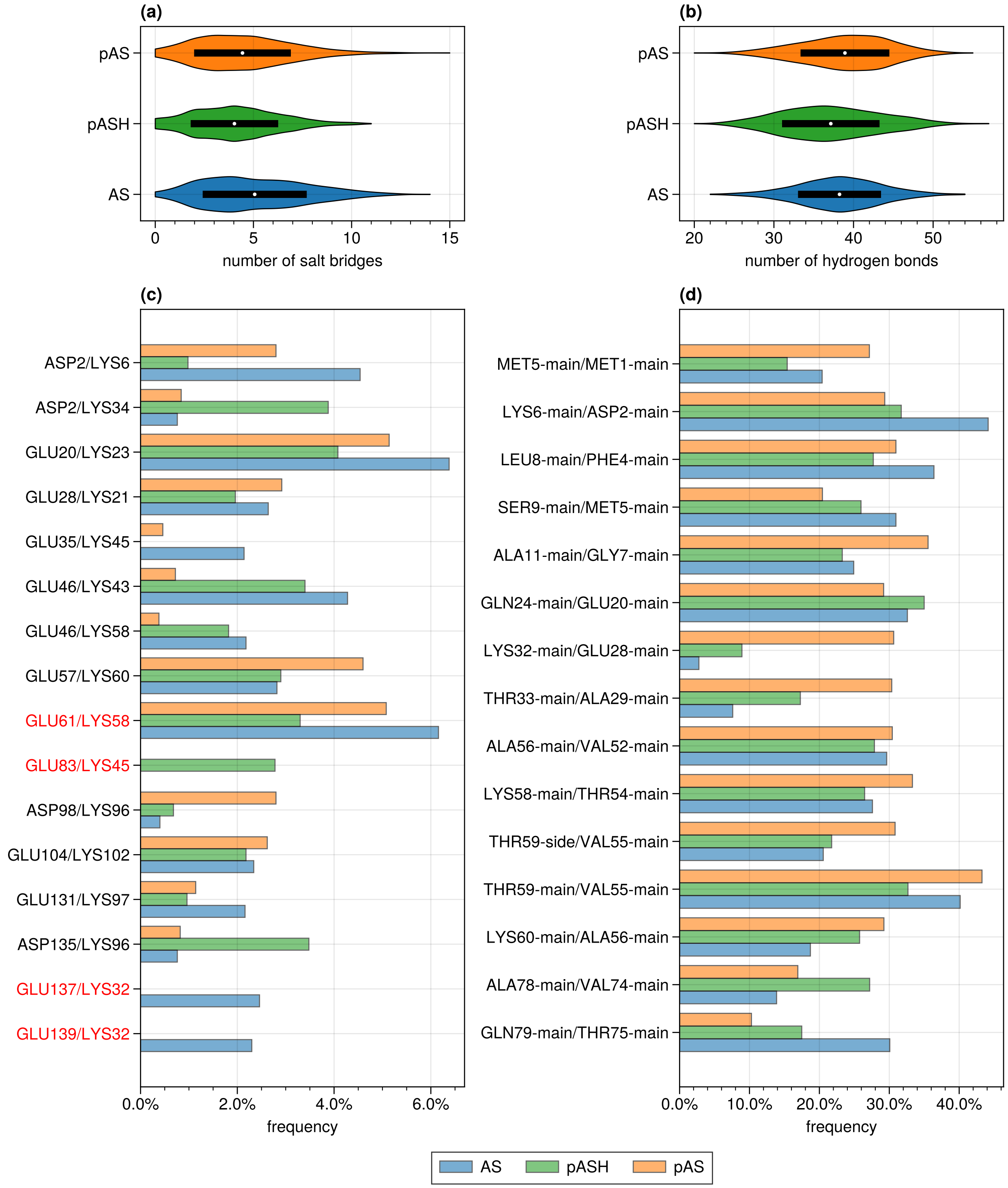

Figure S17: Salt bridges and hydrogen bonds of AS (blue), pASH (green) and pAS (orange). We report the interactions occuring during at least 2 % and 25 % of the converged trajectory, respectively. (a-b): Distribution of the salt bridges (a) and hydrogen bonds (b). (c-d): Frequency with which intradomain (black labels) and interdomain (red labels) salt bridges (c) and hydrogen bonds (d) are found in the three proteins.

Ramachandran plot. The Ramachandran plots (Figure S18) show no discernible differences and exhibit allowed ϕ and Ψ values.

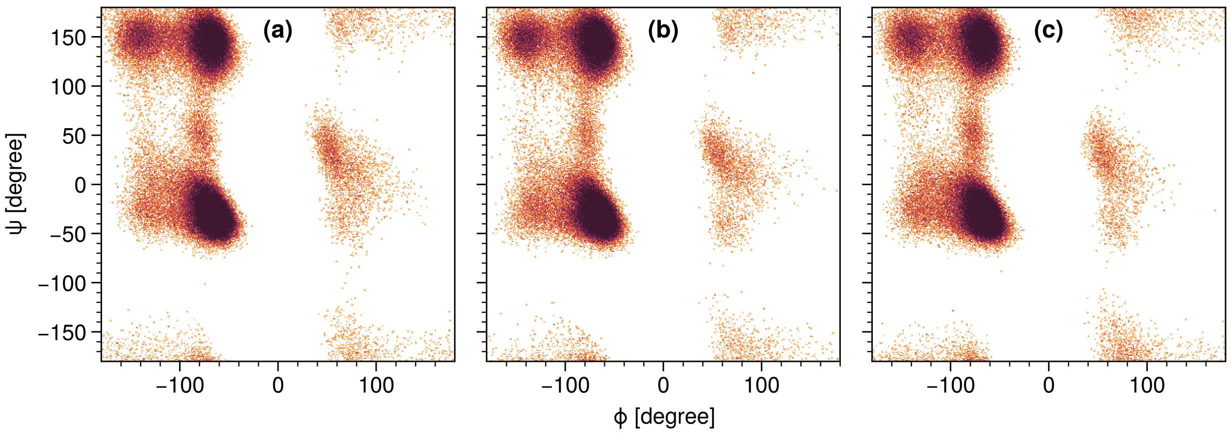

Figure S18: Ramachandran plots for all (a) AS, (b) pASH and (c) pAS from the a99SB-*disp* simulation.

Radial distribution functions. This section covers additional RDFs from the a99SB-*disp* simulations.

Some RDFs (Figure S19) did not converge to 1, because the system was not isotropic. The coordination number reads:

$$N(r) = \rho \cdot 4\pi \int_0^r g(r) r^2 \, \mathrm{d}r \tag{1}$$

with ρ being the density of atoms in the simulation box. The integrals are shown up to 0.26 nm (Figure S20) as this covers the peaks visible in Figure S19 which can be attributed to hydrogen bonds.

To eliminate the possibility of double counting solvent molecules, the RDF and integral in Figure S21 show that there are on average 12 solvent molecules surrounding the phosphoryl group in pAS and a slightly reduced 9 around pASH. This is more than the expected maximum of 3 solvent molecules that can be bound via hydrogen bonding per oxygen atom. This was confirmed through visual inspection along the converged part of the trajectories. This over-hydration is attributable to either the high charge density at the site, or an insufficient description of the hydration of phosphoryl moieties by the TIP4P-D water model[4] employed in the current study.

Overall, the a99SB-*disp* force field based simulations show markedly more artefacts in the phosphate hydration than those based on the DES-Amber force field.

5 AS with protonated phosphate in pSer129 (pASH)

All results in this section used the a99SB-*disp* force field simulations, following exactly the same protocol as that of pAS and AS, but only running 25 ns of REST2 simulations. Thus,

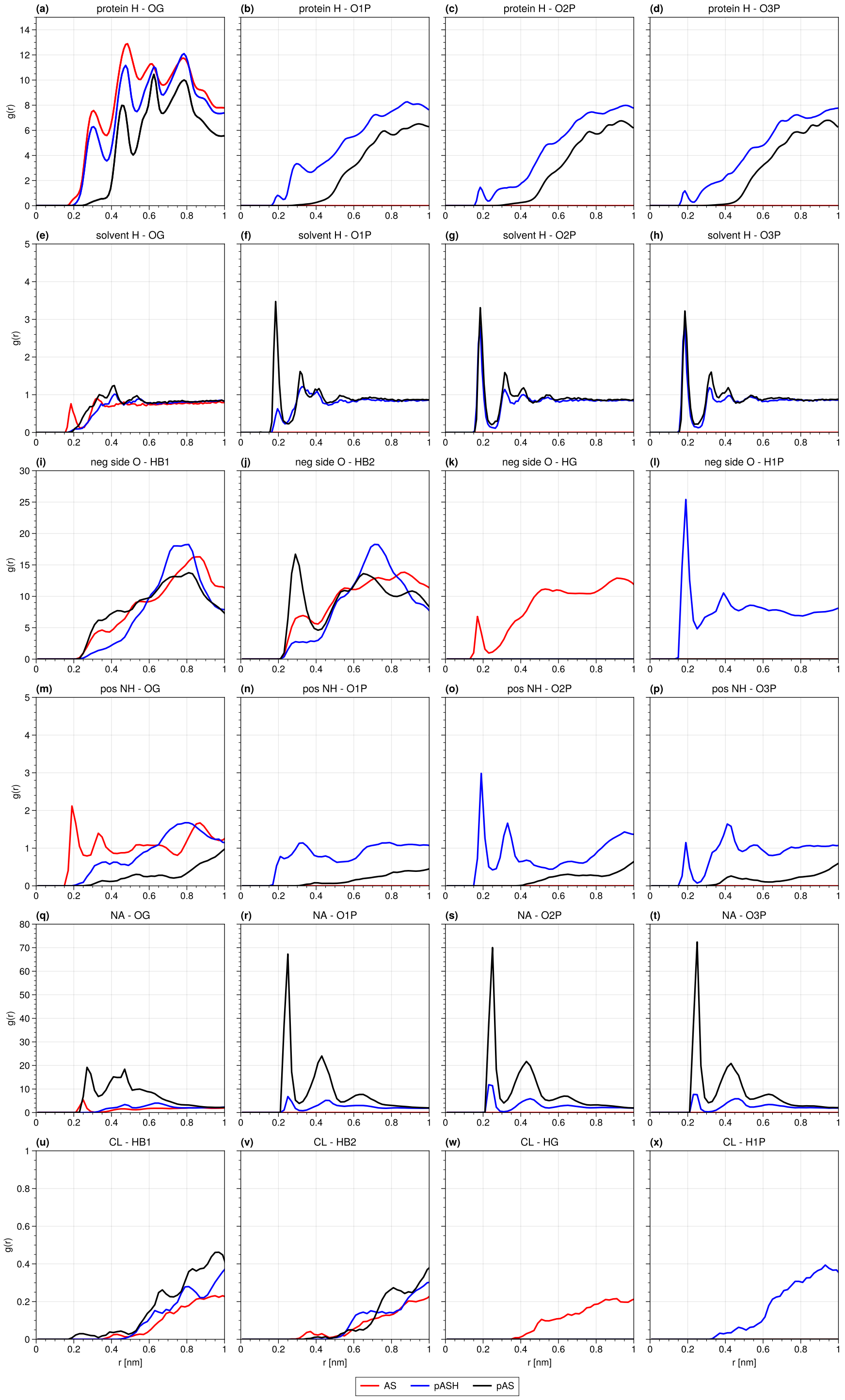

Figure S19: a99SB-*disp* simulation: RDF $g(r)$ of the oxygen atoms of residue 129 side-chain and hydrogen atoms bonded to polar atoms of all other residues (a-d), water hydrogens (e-h), hydrogens atoms bonded to positively charged nitrogen (m-p), and sodium ions (q-t) and RDF of hydrogen atoms of residue 129 side-chain and negatively charged oxygens of all other residues (i-l) and chlorine ions (u-x). (a), (e), (m), (q) compare the RDF for pAS (black) and AS (red) for the oxygen atom in the side-chain. (b-d), (f-h), (n-p), (r-t) illustrate the rdf for the terminal phosphate oxygens. (i-j), (u-v) compare the rdf of pAS (black) and AS (red) for the side-chain hydrogens bonded to carbon. (k), (w) show the rdf of the hydroxyl group. (l), (x) show the rdf of the monoprotonated phosphoryl group.

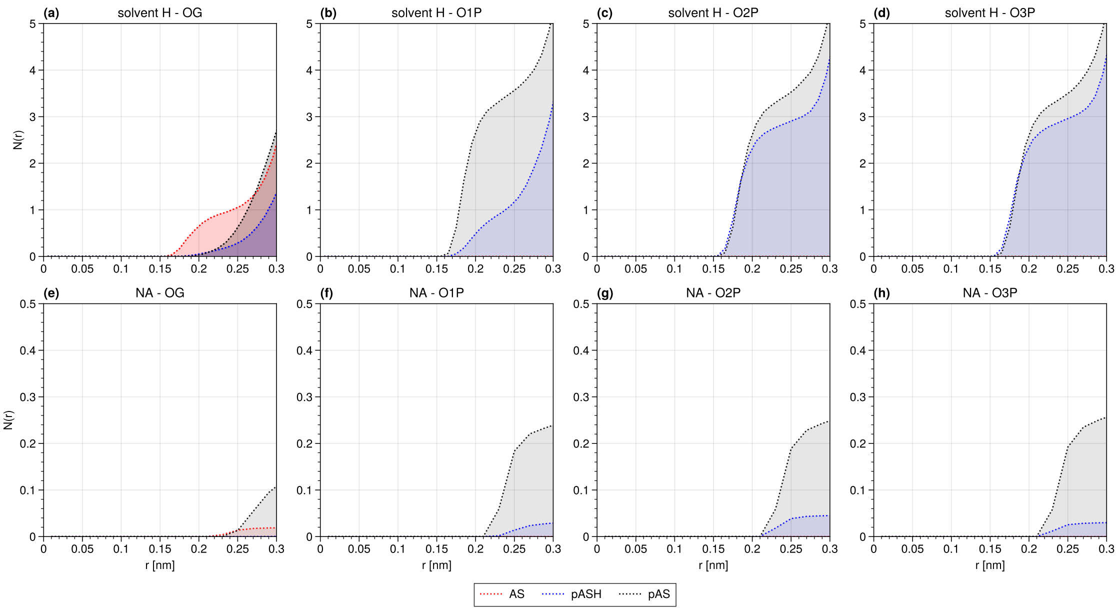

Figure S20: Spherical integrals of the RDFs presented in Figure S19. (a-d) show the integrals of (e-h) in Figure S19 and (e-h) who the integrals of (q-t) in Figure S19. The other integrals are not shown as they are almost zero.

quantitative comparisons are only made with the 25 ns a99SB-*disp* simulations of pAS and AS. The results for the latter are qualitatively similar to those obtained with the 500 ns REST2 simulations using the DES-Amber force field, but obviously differ quantitatively.

Both the radius of gyration and the hydrodynamic radius (Table S9), the SASA (Table S11) and RDFs (Figure S19) increase on passing from AS to pASH to pAS. The number of hydrogen bonds and salt bridges of pASH (Table S9) are lower than those of pAS. The contact maps of pAS and pASH (Figure S22) are similar. The N-terminus/C-terminus distance (Table S10) and the Ramachandran plot (Figure S18) are similar to those of AS and pAS.

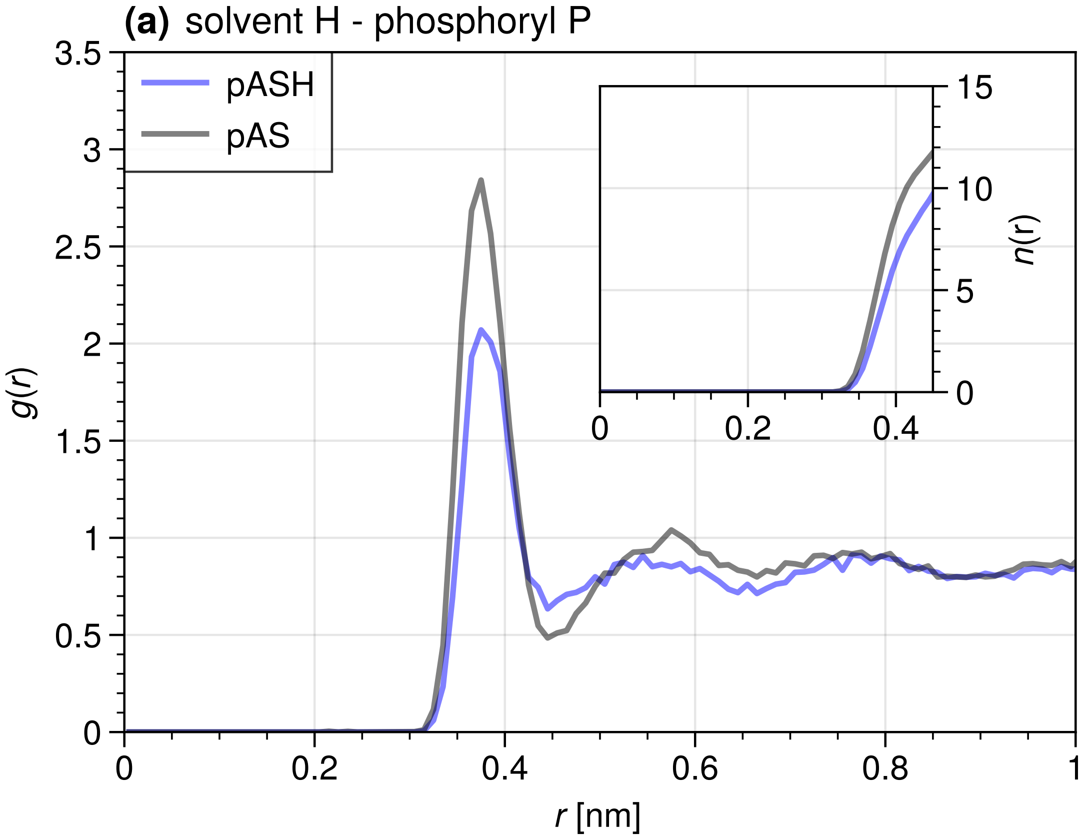

Figure S21: a99SB-*disp* simulation: RDF $g(r)$ of the phosphoryl phosphor atom and solvent hydrogen atoms. The inset shows the cumulative integral $n(r)$ of $g(r)$ up to 4.5 nm (the end of the first solvation shell/peak around the three phosphoryl terminal oxygens).

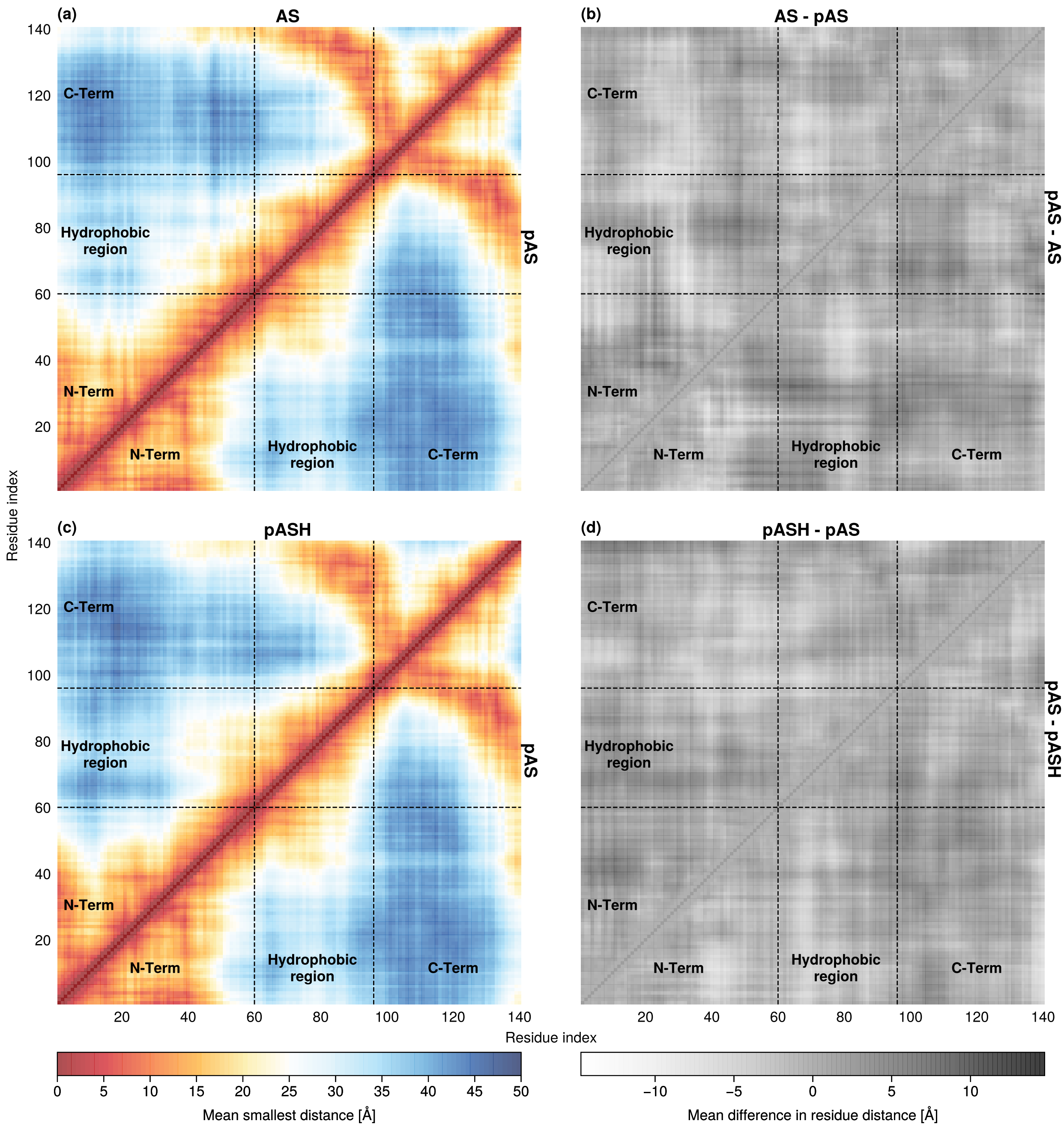

Figure S22: Contact maps of AS (a, triangle above) and pASH (c, triangle above) contrasted with pAS (a,c, triangle below). Mean residue distance differences between AS (b, triangle above) and pASH (d, triangle above) with pAS (b, d, triangle below).

The trajectories of pASH and pAS simulations are broadly similar (Figures S17 to S22). The most notable exception is in the location of interdomain hydrogen bonds (Figure S17(c)). These differing locations are likely an artefact of insufficient sampling, as the number of intermolecular hydrogen bonds does not change among the two proteins. The phosphoryl group is as (slightly less) solvated when singly protonated (pASH) as AS (than in) pAS (Figure S20). In both pASH and pAS, S129 is not involved in salt bridges or hydrogen bonding (Fig-

ure S17). The contact maps of pASH and pAS (Figure S22) show no significant differences. We conclude that if pASH proteins are present at physiological pH, they contribute to the conformational properties of the protein similarly to those of pAS.

6 Secondary Structures of AS and pAS

(i) Secondary structure elements over the last 500 ns of REST2 simulations were identified using the DSSP code.[5,6] and (ii), specifically for the β-hairpin-like in the hydrophobic region, the criterion of ref. 7. This secondary structure is assured to be formed if the RMSD between 12 residues in the hydrophobic region.[2] and a reference β-hairpin is defined as residues 36-47 of the antimicrobial peptide Arenicin-2 dimer (PDBid: `2L8X`) is 2.5 Å or lower.

(i) The number of loops and irregular elements turned out to depend largely on the type of force field (Figure S23(a-b)). The DES-Amber force field-based simulation exhibit a larger difference in α-helix, irregular and loop element, hydrogen bonded turn and bend content than the simulations based on a99SB-*disp* force field. However, the changes upon phosphorylation in the monomer are more pronounced in the hydrophobic region, regardless of the force field.

(ii) Similar to (i), the content of β-hairpin-like structures turns out to depend strongly on the force field chosen (Figure S23(c-d)). The simulations based on DES-Amber exhibit elevated content around residues 61-73. Except for pASH, the simulations based on a99SB-*disp* display notably less content of these structures overall. The pASH simulation also displays higher content of β-hairpin-like structures around residues 72-84.

We finally investigate the impact of the criterion used to define the β-hairpin. By using RMSD $\leq$ 2.0 Å, the content turns out to reduce dramatically to 2 %. Thus, the impact of the criterion used on the results is very large.

[2]For each window, the simulation trajectories were aligned using the MDAnalysis code.[8–11]

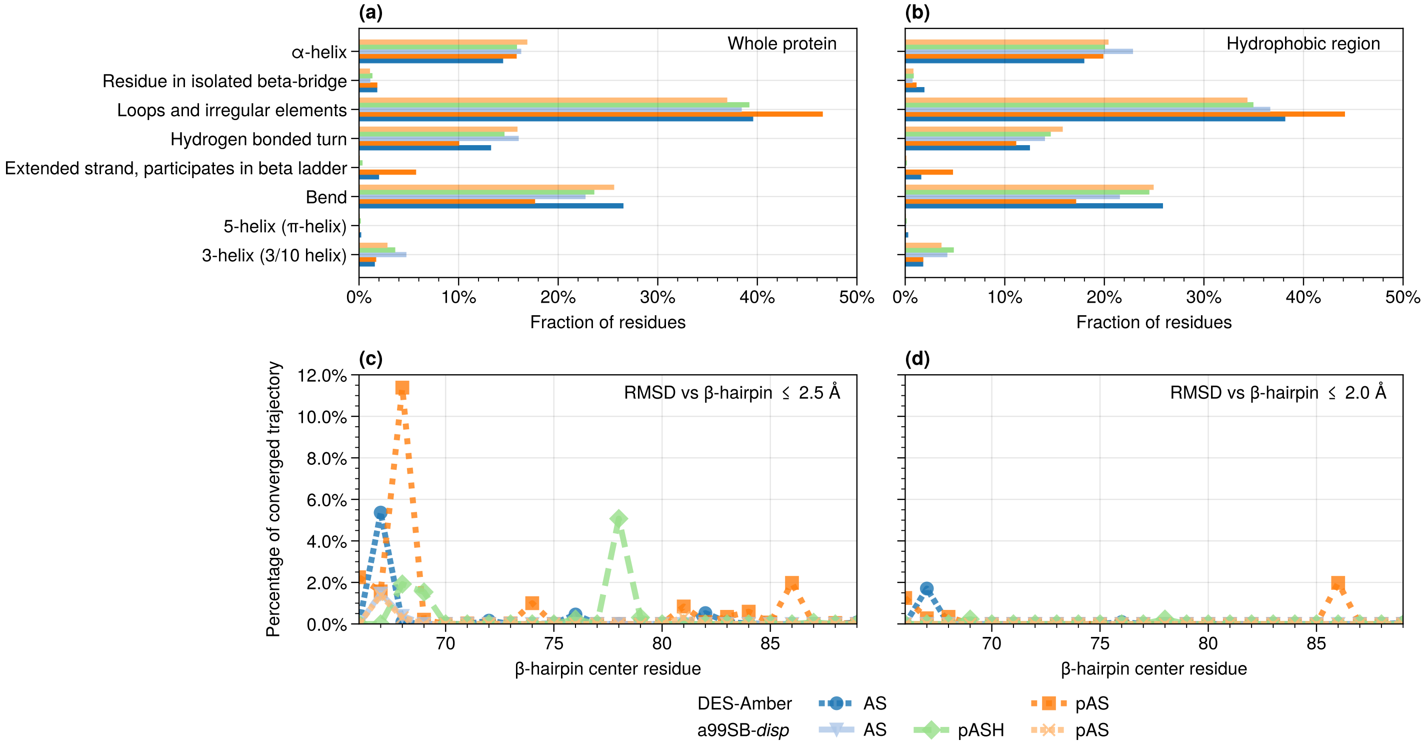

Figure S23: Secondary structure content identified by the DSSP code[5,6] for the whole protein (a) and only the hydrophobic region (b). Percentage of analyzed frames within an RMSD cut-offs of 2.5 Å (c) and 2.0 Å of PDB structure 2L8X for mid-points of 12 residue sliding windows.

References

(1) Wang, L.; Friesner, R. A.; Berne, B. Replica exchange with solute scaling: a more efficient version of replica exchange with solute tempering (REST2). *The Journal of Physical Chemistry B* **2011**, *115*, 9431–9438.

(2) Appadurai, R.; Koneru, J. K.; Bonomi, M.; Robustelli, P.; Srivastava, A. Clustering Heterogeneous Conformational Ensembles of Intrinsically Disordered Proteins With T-Distributed Stochastic Neighbor Embedding. *Journal of Chemical Theory and Computation* **2023**, *19*, 4711–4727.

(3) Maltsev, A. S.; Ying, J.; Bax, A. Impact of N-terminal acetylation of α-synuclein on its random coil and lipid binding properties. *Biochemistry* **2012**, *51*, 5004–5013.

(4) Robustelli, P.; Piana, S.; Shaw, D. E. Developing a molecular dynamics force field for both folded and disordered protein states. *Proceedings of the National Academy of Sciences* **2018**, *115*, E4758–E4766.

(5) Kabsch, W.; Sander, C. Dictionary of protein secondary structure: pattern recogni-

tion of hydrogen-bonded and geometrical features. *Biopolymers: Original Research on Biomolecules* **1983**, *22*, 2577–2637.

(6) Touw, W. G.; Baakman, C.; Black, J.; te Beek, T. A. H.; Krieger, E.; Joosten, R. P.; Vriend, G. A Series of Pdb-Related Databanks for Everyday Needs. *Nucleic Acids Research* **2014**, *43*, D364–D368.

(7) Semenyuk, P. I. Alpha-Synuclein Phosphorylation Induces Amyloid Conversion Via Enhanced Electrostatic Bridging: Insights From Molecular Modeling of the Full-Length Protein. *Biophysical Chemistry* **2024**, *307*, 107196.

(8) Gowers, R.; Linke, M.; Barnoud, J.; Reddy, T.; Melo, M.; Seyler, S.; Domaski, J.; Dotson, D.; Buchoux, S.; Kenney, I.; Beckstein, O. MDAnalysis: A Python Package for the Rapid Analysis of Molecular Dynamics Simulations. Proceedings of the Python in Science Conference. 2016; p nil.

(9) MichaudAgrawal, N.; Denning, E. J.; Woolf, T. B.; Beckstein, O. Mdanalysis: a Toolkit for the Analysis of Molecular Dynamics Simulations. *Journal of Computational Chemistry* **2011**, *32*, 2319–2327.

(10) Theobald, D. L. Rapid Calculation of Rmsds Using a Quaternion-Based Characteristic Polynomial. *Acta Crystallographica Section A Foundations of Crystallography* **2005**, *61*, 478–480.

(11) Liu, P.; Agrafiotis, D. K.; Theobald, D. L. Fast Determination of the Optimal Rotational Matrix for Macromolecular Superpositions. *Journal of Computational Chemistry* **2009**, *31*, 1561–1563.
